# Supplementary material for: Primary cilia control cell alignment and patterning in bone development via ceramide-PKCζ-β-catenin signaling
Source: Commun Biol. 2020 Jan 27;3:45. doi: 10.1038/s42003-020-0767-x (PMC6985158; doi:10.1038/s42003-020-0767-x)
Supplement: Supplementary file 1 — Supplementary Information [file 42003_2020_767_MOESM1_ESM.pdf]

Supplementary 1

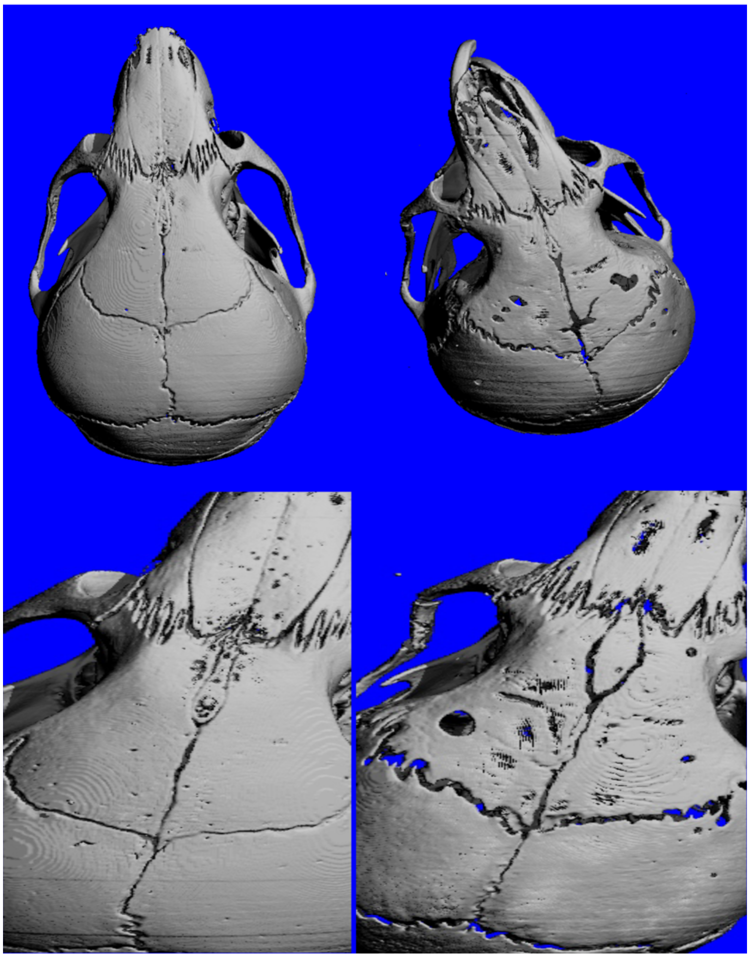

Supplementary Figure 1. Deletion of IFT20 in osteoblast precursor impacts the calvaria and sutures. Micrographs of 3D-reconstructed μCT skull representing 3-month old head dissected from paraformaldehyde fixed OSX-cre controls and IFT20f/f OSX-cre. Upper panel represent a phenotype of bone loss in calvaria and defect in suture formation in addition to malocclusion. Lower panel magnifies the frontal and parietal bones showing abnormal coronal and lambdoid sutures.

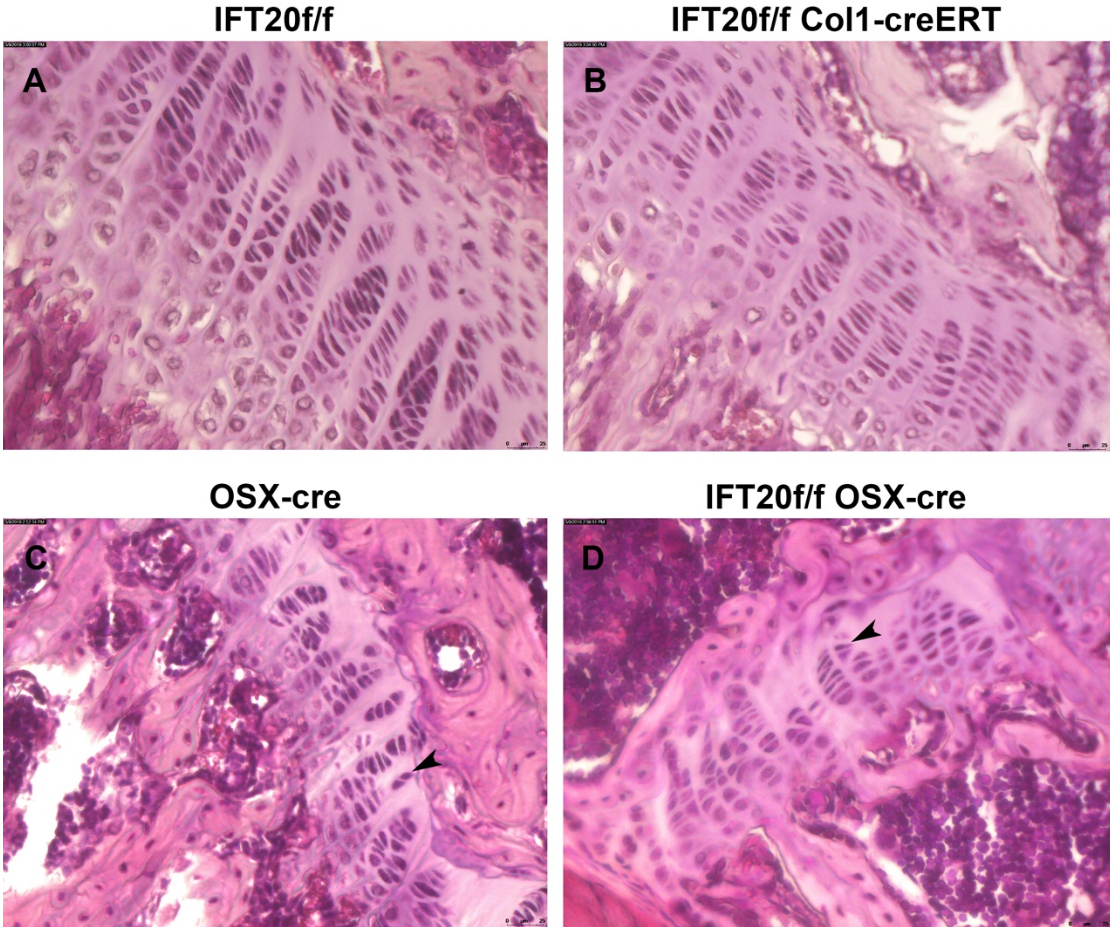

Supplementary Figure 2. Reduced thickness of growth plates at both proliferative and hypertrophic zones in IFT20 deleted proximal tibia. H&E-stained sections of the growth plate in the proximal tibia. Left panels represent the control while right panels represent IFT20 deleted animals. Upper panels represent 1-month old while lower panels represent 3-month old growth plates.

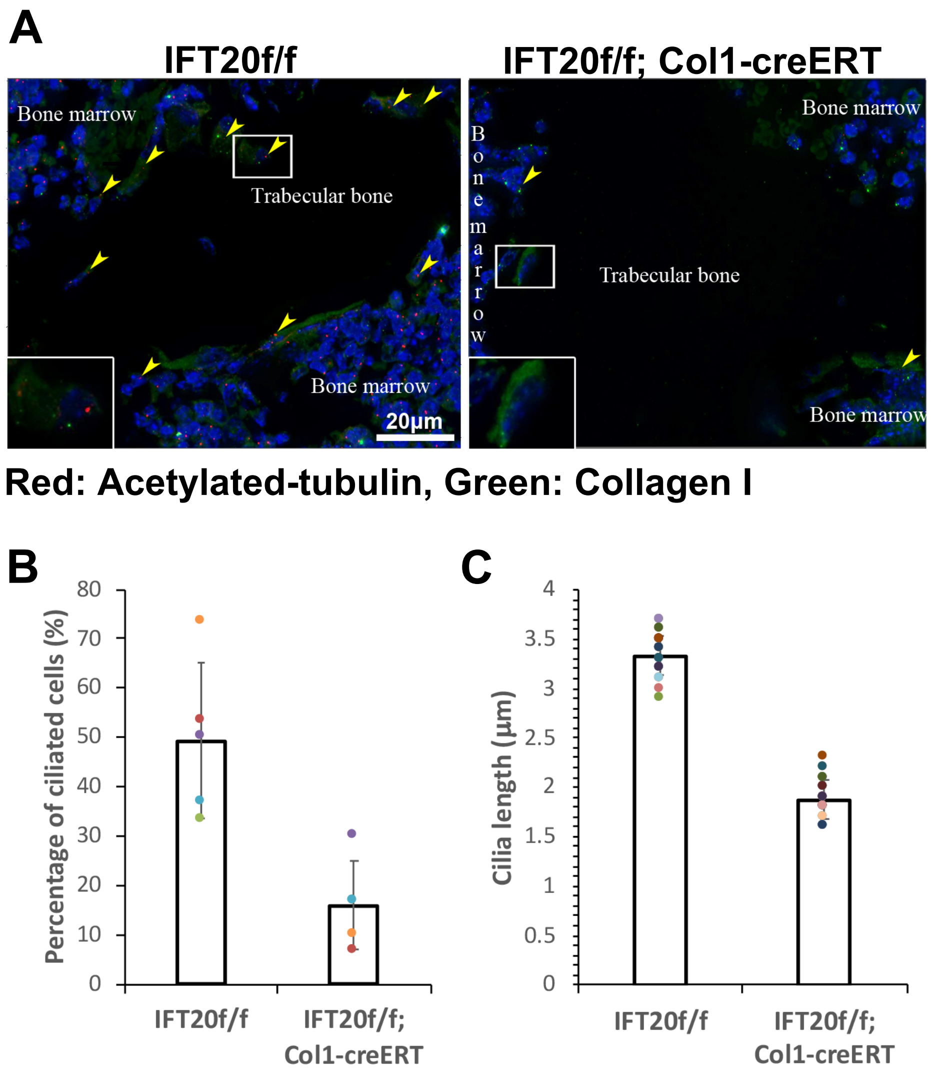

Supplementary Figure 3. Deletion of IFT20 in osteoblasts causes cilia loss.

Micrographs representing Z-stacked 3D-deconvolution processed images captured from the cells aligned on trabecular bone of postnatal day 11 IFT20f/f and IFT20f/f;Col1-creERT femur using Leica DMI6000 inverted epifluorescence microscope under 40X lens (A). Red fluorescent signals detected immunofluorescent staining of Acetylated-tubulin and green fluorescent probed Collagen I and DAPI was depicted in blue. Quatitative comparisons of the primary cilia containing cells percentage (n=3) (B) and the primary cilia length (n=27) (C) in osteoblast.

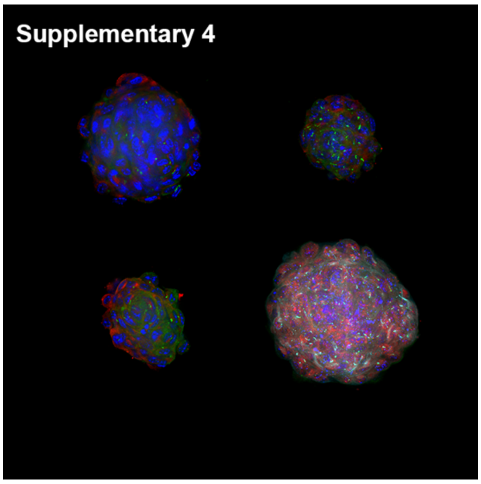

Supplementary Figure 4. Primary cilia in 3 -dimensional spheroids. Primary osteoblasts isolated from cilia GFP mouse were subjected to Ad-creRGD treatment to reveal GFP fluorescence in primary cilia before seeded onto low adherent culture plates to achieve spheroid formation in 24 hours. Paraformaldehyde fixed spheroids were embedded in O.C.T. and subsequently cryosections of 16 μm and immunofluorescently stained for IFT20 (red) and DAPI (blue).

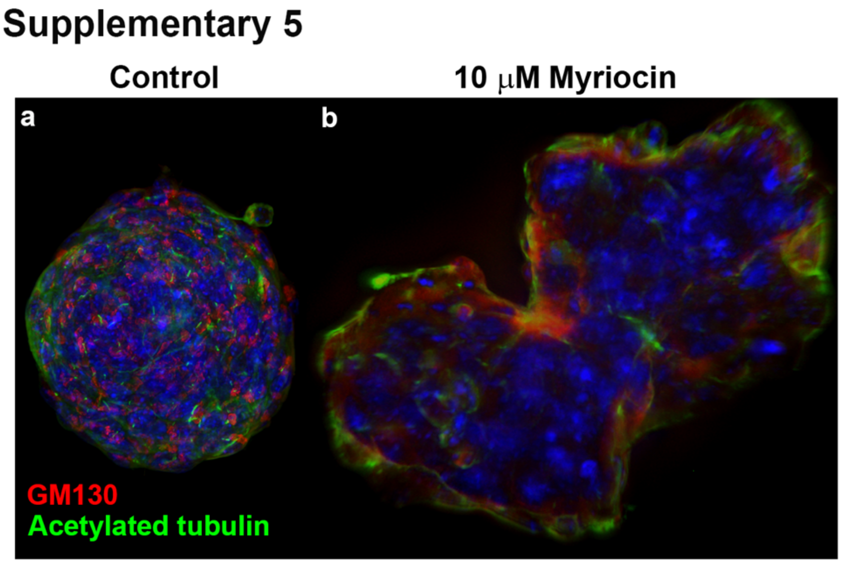

Supplementary Figure 5. Inhibition of Ceramide disrupts cell arrangement in 3D-spheroid . Cells were either treated by DMSO (**a**) or by Ceramide inhibitor Myriocin (10 μM) (**b**) for overnight. Subsequently cells were subjected to spheroid culture and immunofluorescence detecting primary cilia by anti-acetylated-tubulin antibodies and Golgi marker anti-GM130 antibodies (**a,b**).

Supplementary 6

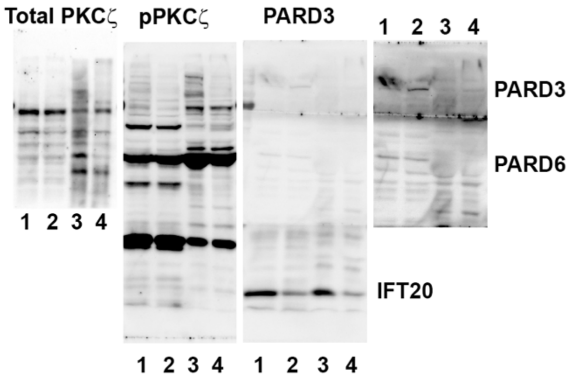

Immunoprecipitation (IP) of Ceramide and Western blots for PKCζ, phosphorylated PKCζ, PARD3, PARD6 and IFT20.

Loading sequence: 1, Total lysates of IFT20f/f POB treated by Ad-null; 2, Total lysates of IFT20f/f treated by Ad-creRGD; 3, IP anti-Ceramide in lysates treated by Ad-null; 4, IP anti-Ceramide in lysates treated by Ad-creRGD.

Supplementary Figure 6. Full blots of raw data for Western analysis of immunoprecipitation in Fig 6m.

Supplementary 7

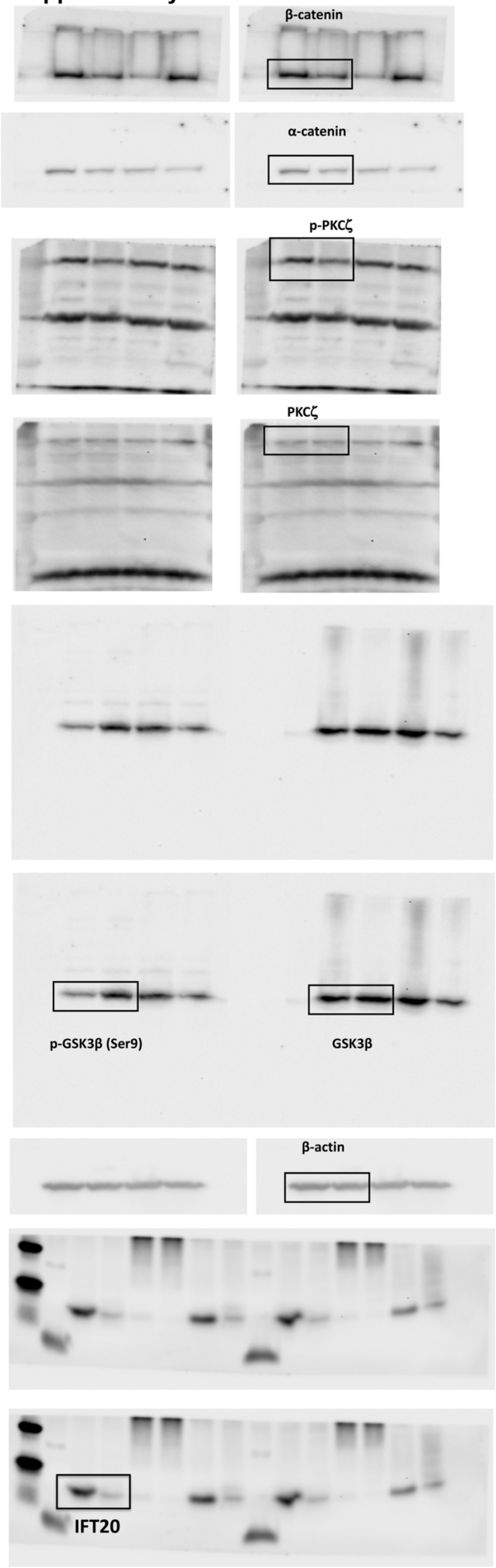

Supplementary Figure 7. Full blots of raw data for Western analysis in Fig 6n.

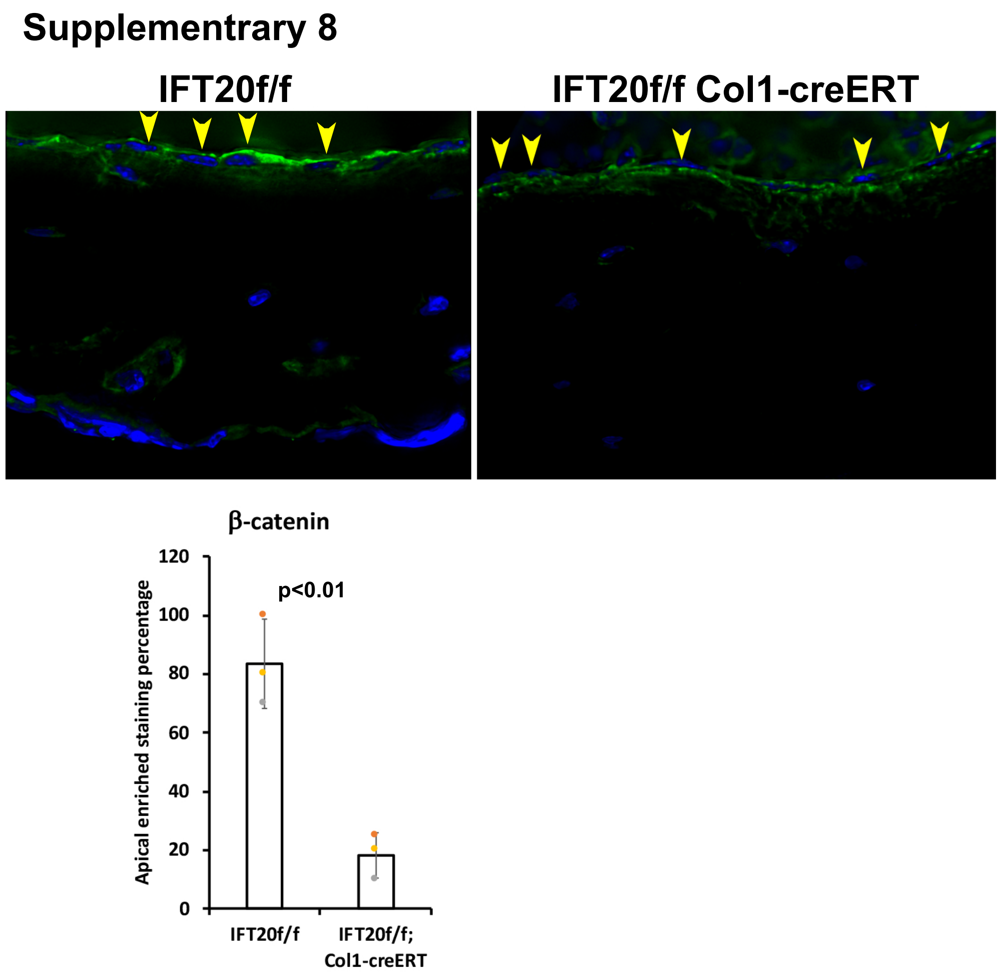

Supplementary Figure 8. Deletion of IFT20 abolishs apical enriched pattern of β-catenin in osteoblasts. Immunofluorescent staining of β-catenin (green) was performed using the cryosections of tibia isolated from IFT20f/f control mouse and IFT20f/f Col1-creERT mouse of one month old. Cells were counted by using DAPI stained nuclei, as indicated by yellow arrow heads.

Supplementary 9

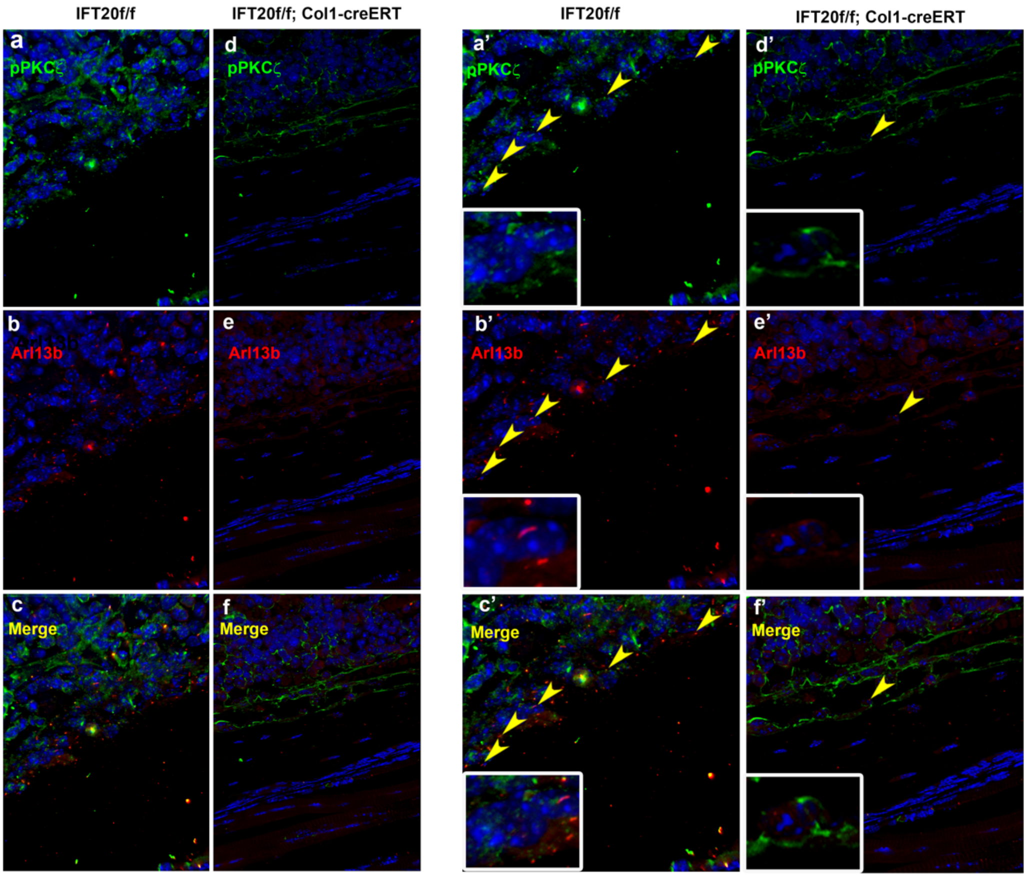

Supplementary Figure 9. Deletion of IFT20 abolishes colocalization of pPKCζ with Arl13b in primary cilia in the osteoblasts lined on the endosteal of cortical bones. Immunofluorescence of pPKCζ (green) and Arl13b (red) in the 14 days pups of IFT20f/f control (a,b,c;a',b',c') on the left and IFT20f/f Col1-creERT (d,e,f;d',e',f') on the right.

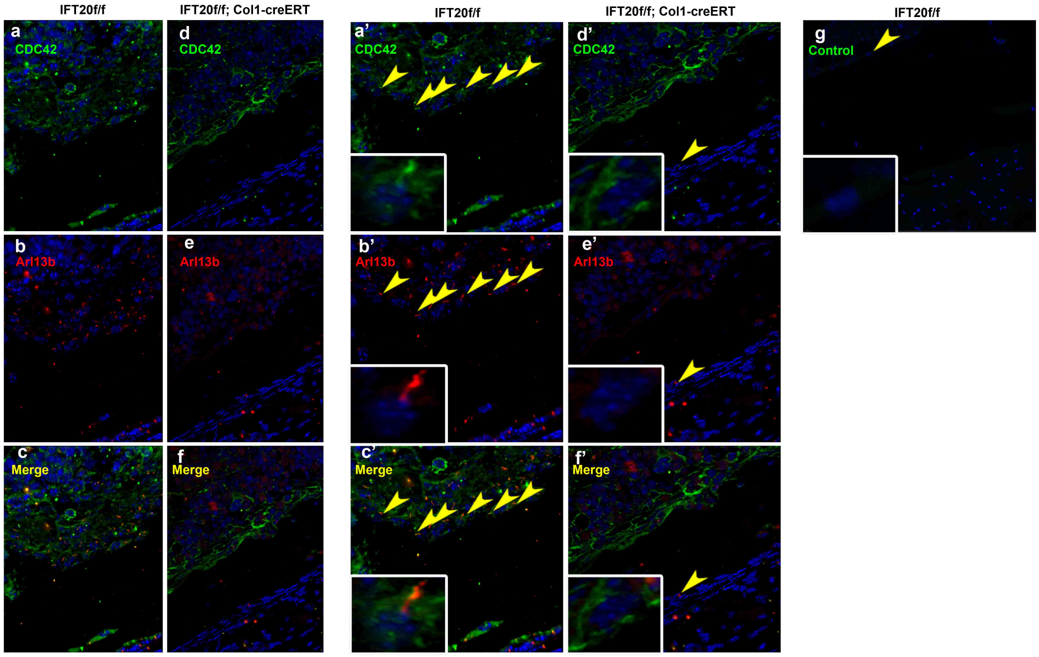

Supplementary Figure 10. Deletion of IFT20 abolishes colocalization of CDC42 with Arl13b in primary cilia of the osteoblasts lined on the endosteal of cortical b.

Immunofluorescence of CDC42 (green) and Arl13b (red) in the 14 days pups of IFT20f/f control on the left (a,b,c;a',b',c') and IFT20f/f Col1-creERT (d,e,f;d',e',f') on the right. g: a negative control for Cdc42 staining.

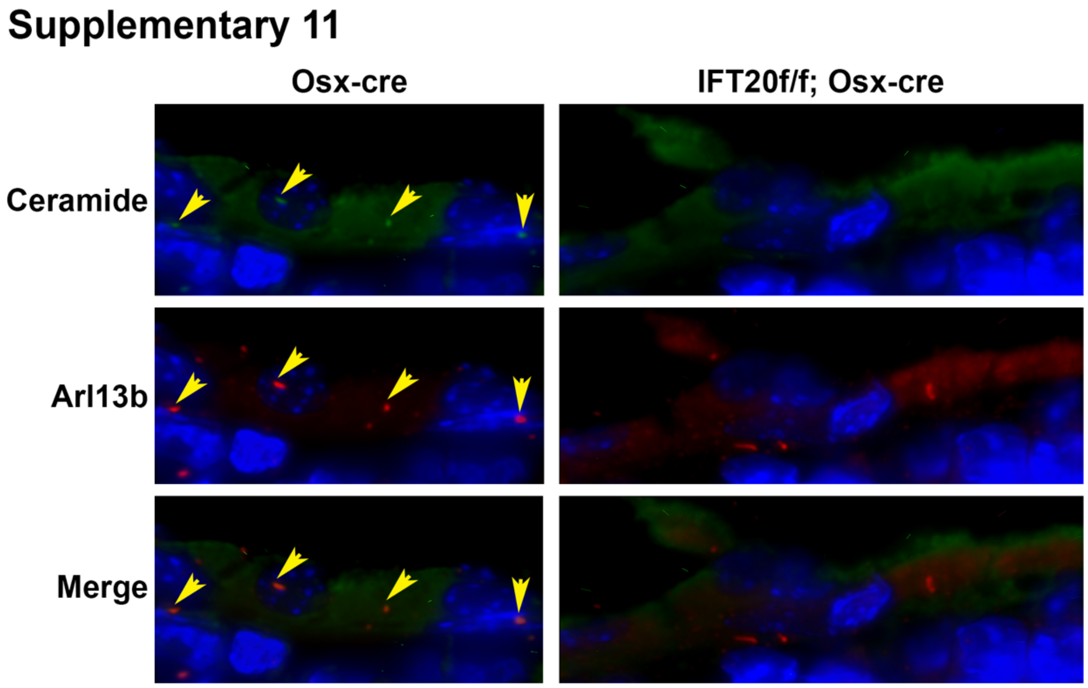

Supplementary Figure 11. Deletion of IFT20 abolishes colocalization of Ceramide with Arl13b in primary cilia of the osteoblasts lined on the endosteal of cortical bones. Immunofluorescence of Ceramide (green) and Arl13b (red) in the 3 months old mice of OSX-cre control on the left and IFT20f/f OSX-cre on the right. Note that in this case some cells show primary cilia with Arl13b (red) staining in the IFT20 deleted animal, however Ceramide (green) is not as intensely stained in the primary cilia as in the control (yellow arrows).

Supplementary 12

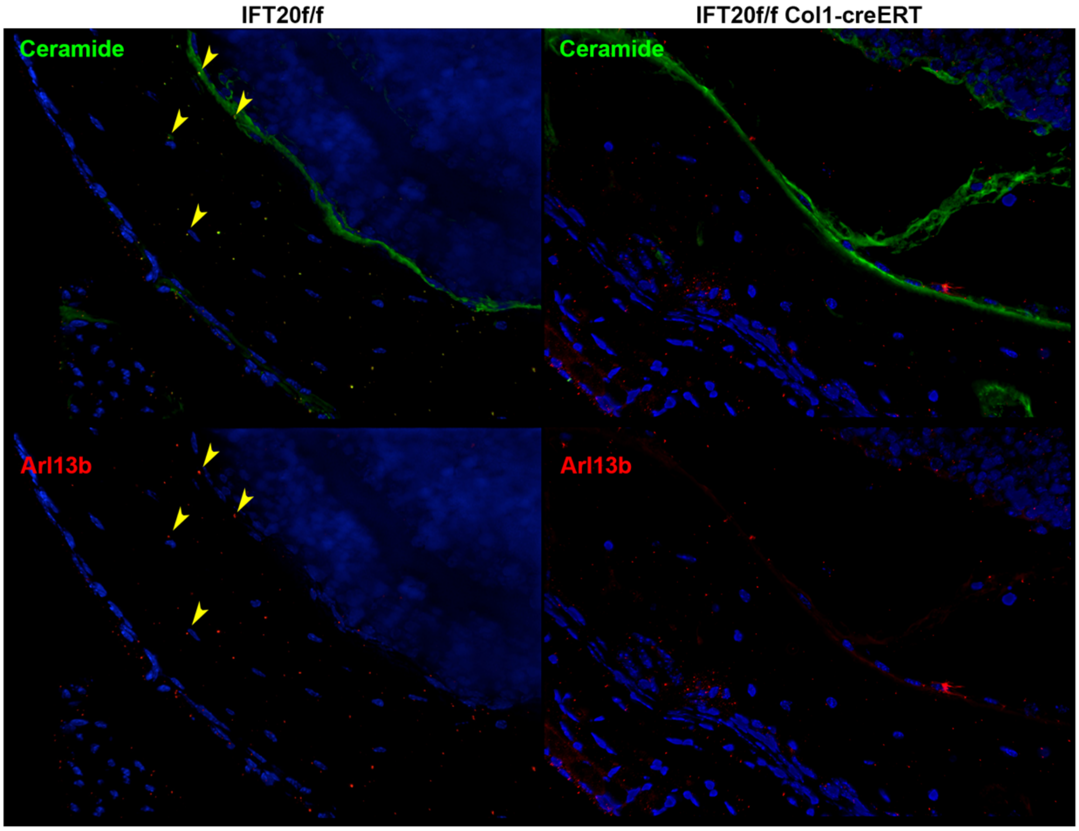

Supplementary Figure 12. Deletion of IFT20 abolishes colocalization of Ceramide with Arl13b in primary cilia in the osteocytes of cortical bones.

Immunofluorescence of Ceramide (green) and Arl13b (red) in the 4 weeks old mice of IFT20f/f control on the left and IFT20f/f Col1-creERT on the right. Ceramide staining (green) coincident with Arl13b signals (red) appear as very short cilia or almost dots as indicated by yellow arrows in the cortical bone whereas the green and red colors do not colocalize in the IFT20 deleted animal.

Supplementary 13

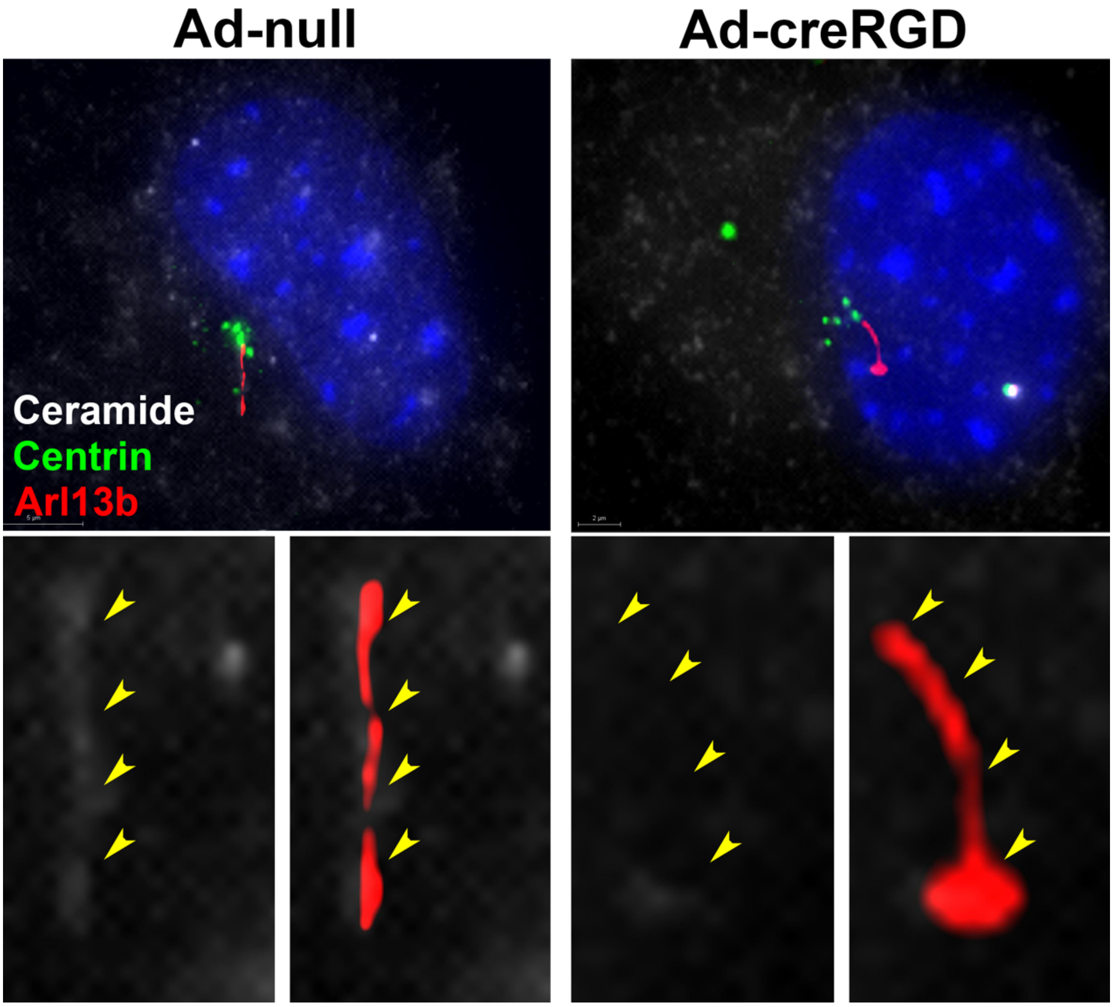

Supplementary Figure 13. Colocalization of Ceramide with Arl13b in the cilia of primary osteoblasts is abolished in IFT20 deleted cells. Reporter mice carry EGFP Centrin2 (green) and Acetylated-tubulin mCherry (red), which allow visualization of basal body and primary cilia respectively are used to cross breed with IFT20f/f. Primary osteoblasts isolated from the neonatal calvaria were treated with either Ad-null for the control or Ad-creRGD for the deletion of IFT20 gene. Ceramide immunofluorescence is represented in grey. In the control cell, Ceramide is detected

in axoneme where IFT20 is present whereas, in some of the cells that is still forming primary cilia in the absence of IFT20, Ceramide is not detected in axoneme.

Supplementary 14

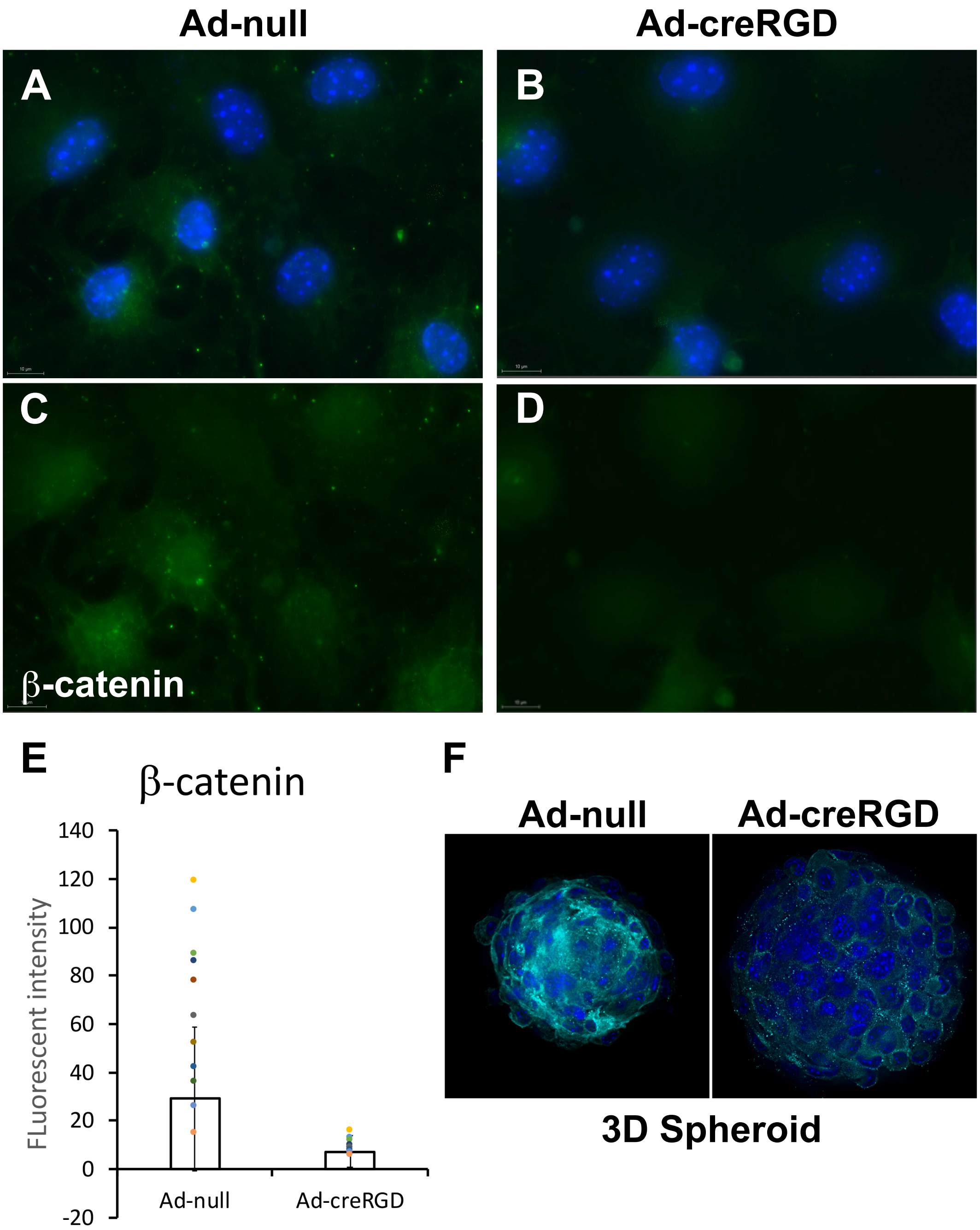

Supplementary Figure 14. Reduced β-catenin fluorescent intensity in IFT20 deficient primary osteoblasts. Primary osteoblasts were isolated from neonatal calvaria and treated with either Ad-null for the control or Ad-creRGD for the deletion of IFT20 gene. β-catenin immunofluorescent staining shows both cytoplasmic and nuclear distribution in the control cells (A, C) and a global reduction of intensity in IFT20 deletion (B, D). Quantification of fluorescent intensity by imageJ shows a significant difference ($p<0.01$). Spheroid culture shows that β-catenin staining pattern changes to membrane when the cells are growing on other cells instead of glass cover slips (F) and again the intensity is reduced with more diffused distribution upon the deletion of IFT20.

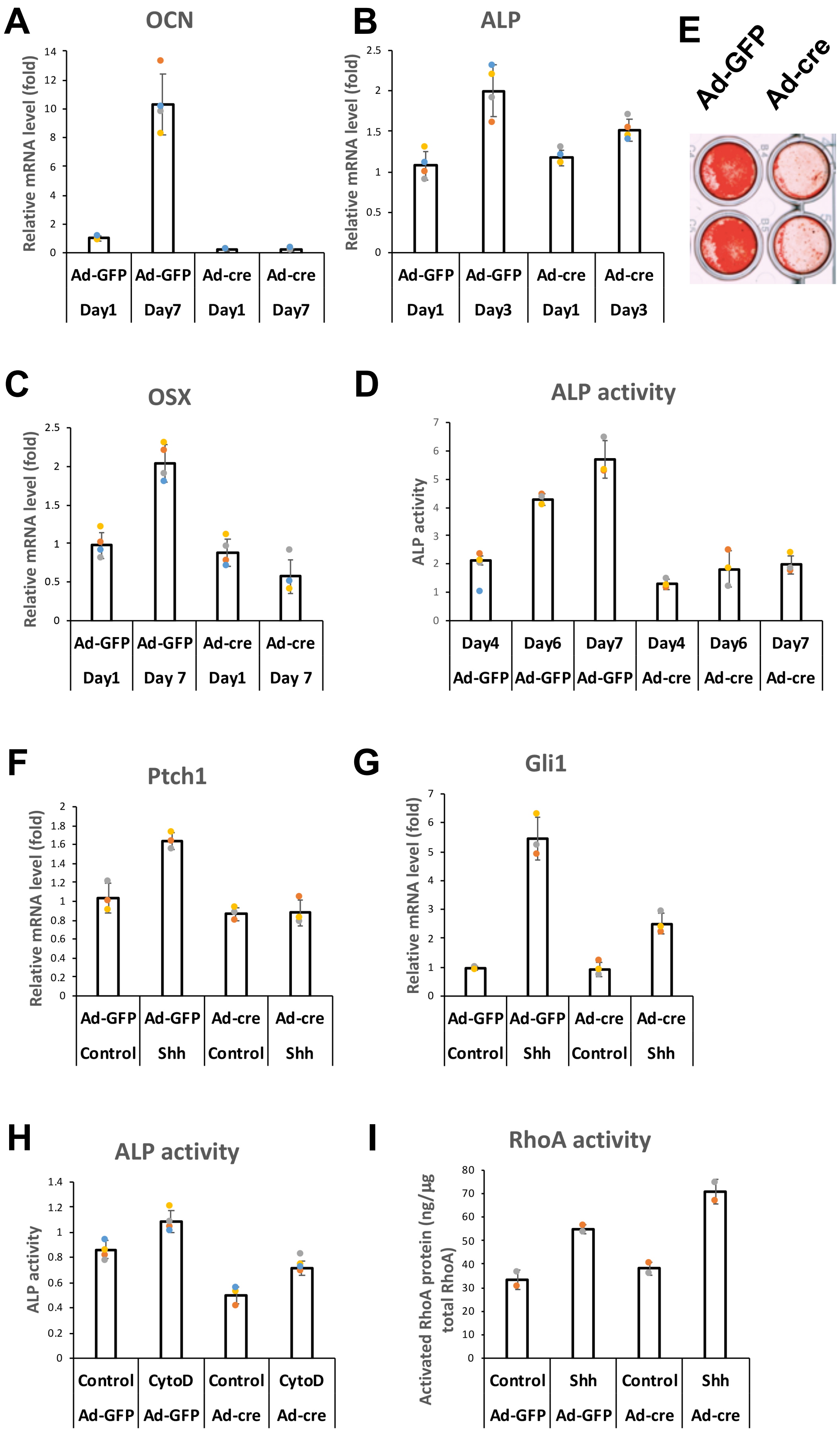
A
OCN
Relative mRNA level (fold)
Ad-GFP Day1
Ad-GFP Day7
Ad-cre Day1
Ad-cre Day7
B
ALP
Relative mRNA level (fold)
Ad-GFP Day1
Ad-GFP Day3
Ad-cre Day1
Ad-cre Day3
E
Ad-GFP
Ad-cre
C
OSX
Relative mRNA level (fold)
Ad-GFP Day1
Ad-GFP Day 7
Ad-cre Day1
Ad-cre Day 7
D
ALP activity
ALP activity
Day4 Ad-GFP
Day6 Ad-GFP
Day7 Ad-GFP
Day4 Ad-cre
Day6 Ad-cre
Day7 Ad-cre
F
Ptch1
Relative mRNA level (fold)
Ad-GFP Control
Ad-GFP Shh
Ad-cre Control
Ad-cre Shh
G
Gli1
Relative mRNA level (fold)
Ad-GFP Control
Ad-GFP Shh
Ad-cre Control
Ad-cre Shh
H
ALP activity
ALP activity
Control Ad-GFP
CytoD Ad-GFP
Control Ad-cre
CytoD Ad-cre
I
RhoA activity
Activated RhoA protein (ng/µg total RhoA)
Control Ad-GFP
Shh Ad-GFP
Control Ad-cre
Shh Ad-cre

Supplementary Figure 15. IFT20 regulates osteogenic differentiation through Hedgehog signaling pathway. Osteocalcin (OCN) transcripts quantified by real-time PCR of the cells in the 1st and 7th day of osteogenic culture (A). Alkaline phosphatase (ALP) transcripts quantified by real-time PCR (B). Osterix (OSX) transcripts quantified by real-time PCR (C). ALP activity (Unit: min^{-1} per mg DNA) during the 4th, 6th and 7th day of cells cultured in in osteogenic media (D). Alizarin red assay performed on the 14th day of osteogenic culture (E). Quantitative PCR of transcriptional targets of Hedgehog signaling in (F) Ptch1 and (G) Gli1. ALP activity affected by actin polymerization inhibitor, cytochalasin D (cytoD) (H). RhoA activity during osteogenesis and Hedgehog stimulation (I).
