# Supplementary material for: Primary cilia control cell alignment and patterning in bone development via ceramide-PKCζ-β-catenin signaling
Source: Commun Biol. 2020 Jan 27;3:45. doi: 10.1038/s42003-020-0767-x (PMC6985158; doi:10.1038/s42003-020-0767-x)
Supplement: Supplementary file 2 — Description of Additional Supplementary Files [file 42003_2020_767_MOESM2_ESM.pdf]

Supplementary data figure legends.

Supplementary data 1: Data source for Figure 1 graphs

Supplementary data 2: Data source for Figure 3 graphs

Supplementary data 3: Data source for Figure 4 graphs

Supplementary data 4: Data source for Figure 5 graphs

Supplementary data 5: Data source for Figure 6 graphs

Supplementary data 6: Data source for Supplementary figure 8 graph

Supplementary data 7: Data source for Supplementary figure 14 graph

Supplementary data 8: Data source for Supplementary figure 15 graphs
