# Supplementary material for: Primary cilia control cell alignment and patterning in bone development via ceramide-PKCζ-β-catenin signaling
Source: Commun Biol. 2020 Jan 27;3:45. doi: 10.1038/s42003-020-0767-x (PMC6985158; doi:10.1038/s42003-020-0767-x)
Supplement: Supplementary file 9 — Supplementary Data 7 [file 42003_2020_767_MOESM9_ESM.pdf]

IFT20wt	Area	Mean	Min	Max		IFT20null
1985	0.003	85.659	9	251		11
2025	0.003	152.893	9	251		5
1300	0.004	14.89	9	119		6
1904	0.009	16.018	9	108		8
1	3.738	13.477	9	107		170
1511	0.011	14.676	9	104		183
835	0.047	11.676	9	89		131
937	2.22E-05	85.5	85	86		38
951	2.22E-05	47.5	9	86		61
954	0.002	10.868	9	86		159
902	1.11E-05	85	85	85		229
972	1.11E-05	85	85	85		120
1696	0.005	15.407	9	80		133
584	2.076	15.756	9	78		168
953	0.003	12.784	9	67		177
2004	0.002	15.503	9	67		198
883	2.22E-05	36	9	63		223
851	1.11E-05	57	57	57		9
822	1.11E-05	52	52	52		18
850	1.11E-05	52	52	52		36
1257	1.11E-05	49	49	49		73
1480	0.002	16.162	9	48		166
764	6.78E-04	15.525	9	46		179
887	5.22E-04	15.851	9	44		193
1239	1.11E-05	42	42	42		196
1221	1.11E-05	39	39	39		7
201	9.56E-04	13.256	9	38		22
704	0.002	12.296	9	38		25
918	1.11E-05	37	37	37		42
855	1.11E-05	36	36	36		47
892	1.11E-05	36	36	36		62
63	0.005	13.266	9	35		69
1243	1.11E-05	35	35	35		71
1266	1.11E-05	35	35	35		74
1592	0.001	12.648	9	35		83
611	0.004	10.42	9	34		84
1906	0.001	13.057	9	34		86
873	1.11E-05	33	33	33		88
1767	6.56E-04	14.186	9	33		89
654	0.003	11.295	9	32		97
784	0.001	12.726	9	32		105
865	2.22E-05	20.5	9	32		106

557	5.56E-04	13.9	9	31	109
856	1.11E-05	31	31	31	112
1101	0.716	11.896	9	31	113
1691	3.56E-04	14.188	9	31	128
817	1.11E-05	30	30	30	154
890	1.11E-05	30	30	30	157
986	3.78E-04	15.353	9	30	160
1210	0.001	11.096	9	30	163
1263	0.003	12.166	9	30	165
1705	0.002	13.222	9	30	169
813	1.11E-05	29	29	29	172
245	7.89E-04	12.577	9	28	174
686	5.89E-04	13.774	9	28	175
219	0.002	11.72	9	27	186
1372	0.001	11.408	9	27	187
1414	2.78E-04	12.76	9	27	189
1437	0.006	10.259	9	27	194
2030	9.00E-04	12.37	9	27	197
193	3.78E-04	12.794	9	26	205
761	1.78E-04	15.625	9	26	208
1202	1.11E-05	26	26	26	213
1331	5.11E-04	12.457	9	25	216
815	2.22E-05	16.5	9	24	219
830	2.22E-05	23.5	23	24	221
906	1.11E-05	24	24	24	224
2021	2.44E-04	13.864	9	24	225
130	0.001	11.431	9	23	227
808	1.11E-05	23	23	23	1
1523	0.013	10.673	9	23	2
2017	1.11E-05	23	23	23	3
965	1.11E-05	22	22	22	4
1027	0.001	10.919	9	22	10
1197	4.44E-05	12.5	9	22	12
2063	2.22E-05	16.5	11	22	13
528	0.003	10.057	9	21	14
737	2.44E-04	12	9	21	15
821	1.11E-05	21	21	21	16
874	1.11E-05	21	21	21	17
891	3.33E-05	13	9	21	19
975	0.016	9.726	9	21	20
1305	1.56E-04	12.929	9	21	21
1658	0.003	10.854	9	21	23
1978	0.001	11.105	9	21	24

2018	2.22E-05	15.5	10	21	26
2020	2.22E-05	21	21	21	27
2022	3.33E-05	17	10	21	28
247	4.22E-04	11.868	9	20	29
538	2.56E-04	12.348	9	20	30
1099	2.89E-04	13.192	9	20	31
1100	0.004	11.081	9	20	32
1566	2.33E-04	12.048	9	20	33
1865	8.33E-04	11.64	9	20	34
802	9.89E-04	12.011	9	19	35
895	0.001	11.764	9	19	37
911	6.56E-04	11.576	9	19	39
1116	3.67E-04	11.485	9	19	40
1282	1.56E-04	13.5	9	19	41
1360	1.11E-04	13	9	19	43
1438	5.11E-04	12.043	9	19	44
1443	0.001	11.009	9	19	45
1582	2.33E-04	11.048	9	19	46
1641	1.67E-04	13	9	19	48
1761	2.44E-04	13.136	9	19	49
163	0.002	10.621	9	18	50
408	2.00E-04	11.5	9	18	51
511	6.67E-04	11.467	9	18	52
554	1.78E-04	11.5	9	18	53
566	0.002	10.408	9	18	54
723	1.11E-05	18	18	18	55
913	4.56E-04	11.878	9	18	56
1198	1.11E-05	18	18	18	57
1422	2.00E-04	12.889	9	18	58
1424	0.002	10.442	9	18	59
1474	0.002	10.153	9	18	60
1923	5.22E-04	11.702	9	18	63
2011	3.33E-05	14.333	12	18	64
2060	4.44E-05	14.75	13	18	65
100	1.78E-04	11.688	9	17	66
310	9.11E-04	10.341	9	17	67
553	5.67E-04	10.451	9	17	68
797	3.33E-05	11.667	9	17	70
809	0.002	10.626	9	17	72
836	1.11E-05	17	17	17	75
857	4.56E-04	10.659	9	17	76
988	3.78E-04	11.441	9	17	77
1214	1.56E-04	12.429	9	17	78

1473	3.11E-04	11.5	9	17	79
1659	0.02	9.805	9	17	80
1711	8.44E-04	10.632	9	17	81
1773	1.56E-04	12.214	9	17	82
1947	2.11E-04	12.474	9	17	85
1980	6.44E-04	11.379	9	17	87
2069	1.11E-05	17	17	17	90
23	0.001	10.398	9	16	91
167	1.00E-04	11.889	9	16	92
338	0.004	10.201	9	16	93
533	4.44E-04	11.15	9	16	94
617	5.11E-04	10.543	9	16	95
790	0.001	10.121	9	16	96
800	2.44E-04	11.773	9	16	98
829	1.11E-05	16	16	16	99
863	1.11E-05	16	16	16	100
926	1.11E-05	16	16	16	101
933	3.33E-05	12.667	9	16	102
981	1.11E-05	16	16	16	103
984	1.11E-05	16	16	16	104
1000	0.04	9.532	9	16	107
1010	1.11E-05	16	16	16	108
1029	5.56E-04	10.04	9	16	110
1148	7.33E-04	10.939	9	16	111
1220	1.11E-05	16	16	16	114
1229	2.11E-04	12	9	16	115
1262	1.11E-05	16	16	16	116
1264	2.33E-04	11.571	9	16	117
1394	1.44E-04	11.846	9	16	118
1526	1.89E-04	11.765	9	16	119
1540	1.67E-04	11.267	9	16	121
1778	3.56E-04	10.594	9	16	122
1828	5.67E-04	11.216	9	16	123
1948	2.44E-04	11.773	9	16	124
1973	7.33E-04	11.03	9	16	125
2012	8.22E-04	10.027	9	16	126
2019	2.22E-05	14	12	16	127
2027	2.22E-05	15	14	16	129
2052	1.11E-05	16	16	16	130
2062	4.44E-05	12.5	9	16	132
58	0.005	9.306	9	15	134
76	0.003	9.672	9	15	135
159	8.67E-04	9.628	9	15	136

260	4.33E-04	10.897	9	15	137
295	6.33E-04	10.386	9	15	138
426	0.005	9.455	9	15	139
539	0.001	9.962	9	15	140
610	4.33E-04	11.154	9	15	141
681	1.22E-04	11.545	9	15	142
715	0.011	9.458	9	15	143
870	1.11E-05	15	15	15	144
879	1.33E-04	11.333	9	15	145
884	1.11E-05	15	15	15	146
903	1.11E-05	15	15	15	147
1217	1.11E-05	15	15	15	148
1457	2.44E-04	11.591	9	15	149
1489	4.89E-04	9.932	9	15	150
1604	4.44E-04	10.375	9	15	151
1804	0.001	10.37	9	15	152
1893	5.67E-04	9.98	9	15	153
1941	0.005	10.073	9	15	155
1976	1.56E-04	11.071	9	15	156
1986	3.33E-05	12	10	15	158
2008	6.67E-05	12.667	9	15	161
2023	4.44E-05	13.5	12	15	162
2032	4.44E-05	13.25	11	15	164
2033	1.11E-05	15	15	15	167
2059	2.22E-05	13.5	12	15	171
42	7.11E-04	9.984	9	14	173
128	3.11E-04	10.393	9	14	176
141	2.89E-04	10.654	9	14	178
280	4.56E-04	9.976	9	14	180
287	3.11E-04	11.286	9	14	181
423	6.11E-04	10.491	9	14	182
433	4.00E-04	10.75	9	14	184
458	3.44E-04	10.645	9	14	185
470	6.22E-04	9.946	9	14	188
492	0.002	9.763	9	14	190
519	0.003	9.723	9	14	191
597	2.78E-04	10.4	9	14	192
612	2.44E-04	10.818	9	14	195
641	2.33E-04	10.857	9	14	199
670	0.002	9.633	9	14	200
683	4.11E-04	11.243	9	14	201
702	2.44E-04	10.364	9	14	202
846	0.011	9.427	9	14	203

897	1.11E-05	14	14	14	204
1216	1.78E-04	10.625	9	14	206
1234	1.11E-05	14	14	14	207
1236	1.89E-04	10.588	9	14	209
1245	1.00E-04	11.222	9	14	210
1283	1.11E-05	14	14	14	211
1389	3.56E-04	10.531	9	14	212
1442	2.67E-04	11.417	10	14	214
1471	2.11E-04	10.684	9	14	215
1514	4.22E-04	10.763	9	14	217
1632	4.11E-04	9.946	9	14	218
1748	0.001	9.898	9	14	220
1857	2.22E-04	10.4	9	14	222
1889	2.00E-04	10.833	9	14	226
1898	0.001	9.716	9	14	228
1966	1.56E-04	10.857	9	14	230
2009	4.44E-05	11.25	10	14	231
2029	2.22E-05	12.5	11	14	232
2045	0.001	10.113	9	14	233
2067	1.11E-05	14	14	14	
2068	1.11E-05	14	14	14	
49	3.00E-04	10.259	9	13	
197	1.33E-04	9.833	9	13	
200	9.33E-04	9.726	9	13	
288	9.78E-04	9.511	9	13	
289	1.33E-04	9.833	9	13	
308	0.003	9.407	9	13	
343	0.002	9.716	9	13	
349	2.56E-04	10.391	9	13	
363	2.11E-04	10.579	9	13	
403	2.11E-04	9.842	9	13	
407	1.33E-04	10.833	9	13	
473	0.003	9.444	9	13	
475	1.67E-04	10.133	9	13	
537	0.002	9.32	9	13	
589	1.11E-04	10.4	9	13	
630	7.78E-05	10.429	9	13	
672	2.89E-04	10.115	9	13	
705	1.11E-05	13	13	13	
724	3.89E-04	10.857	9	13	
740	5.00E-04	10.444	9	13	
793	4.44E-05	10	9	13	
831	1.11E-05	13	13	13	

849	4.11E-04	9.892	9	13
976	4.56E-04	10.293	9	13
979	1.11E-05	13	13	13
991	2.22E-05	11	9	13
1009	3.33E-04	10.033	9	13
1039	8.89E-05	10.875	9	13
1058	1.33E-04	10.583	9	13
1133	4.89E-04	10.25	9	13
1137	2.67E-04	10.375	9	13
1156	3.22E-04	10.034	9	13
1284	2.00E-04	10.222	9	13
1292	4.56E-04	9.78	9	13
1296	1.11E-05	13	13	13
1349	1.33E-04	10.167	9	13
1458	0.002	9.731	9	13
1476	5.78E-04	9.865	9	13
1544	6.44E-04	9.914	9	13
1559	7.89E-04	9.803	9	13
1562	2.44E-04	10.273	9	13
1680	5.22E-04	10.234	9	13
1685	1.67E-04	10.933	9	13
1722	2.33E-04	10.143	9	13
1762	4.00E-04	10	9	13
1794	3.78E-04	10.588	9	13
1833	9.67E-04	10.172	9	13
1869	5.56E-04	9.98	9	13
1871	0.002	9.955	9	13
1888	4.11E-04	10.324	9	13
1905	5.44E-04	10.082	9	13
1907	0.002	10.228	9	13
1938	7.89E-04	10.099	9	13
1942	3.11E-04	10.393	9	13
2010	1.11E-05	13	13	13
2016	1.11E-05	13	13	13
2039	2.44E-04	10.409	9	13
2065	2.22E-05	11.5	10	13
47	1.33E-04	9.917	9	12
61	2.67E-04	10.333	9	12
70	7.78E-05	10.143	9	12
77	0.002	9.423	9	12
106	2.22E-04	9.95	9	12
108	9.78E-04	10.193	9	12
155	8.89E-04	9.588	9	12

161	1.89E-04	9.824	9	12
175	1.44E-04	10.231	9	12
186	1.56E-04	9.714	9	12
198	4.11E-04	9.973	9	12
199	0.005	9.512	9	12
282	2.89E-04	10.038	9	12
311	5.00E-04	9.889	9	12
332	3.22E-04	10.345	9	12
345	0.002	9.311	9	12
372	0.005	9.279	9	12
379	3.89E-04	9.8	9	12
390	2.33E-04	9.81	9	12
411	4.22E-04	9.474	9	12
432	2.22E-04	10.1	9	12
436	7.00E-04	9.778	9	12
440	2.67E-04	10.375	9	12
451	5.56E-04	9.48	9	12
485	1.56E-04	9.929	9	12
503	8.89E-05	10	9	12
555	6.44E-04	9.586	9	12
575	0.004	9.234	9	12
669	2.00E-04	10	9	12
684	5.67E-04	9.882	9	12
698	9.44E-04	9.188	9	12
743	0.004	9.189	9	12
752	4.67E-04	9.786	9	12
772	1.33E-04	10.417	9	12
798	1.11E-05	12	12	12
923	2.22E-04	9.95	9	12
930	2.44E-04	9.818	9	12
955	1.78E-04	10.375	9	12
956	2.11E-04	9.947	9	12
963	1.11E-05	12	12	12
999	1.11E-05	12	12	12
1033	1.11E-05	12	12	12
1135	8.33E-04	9.693	9	12
1161	0.001	9.5	9	12
1186	1.11E-05	12	12	12
1187	1.11E-05	12	12	12
1218	0.002	9.701	9	12
1247	1.11E-05	12	12	12
1249	3.67E-04	9.818	9	12
1251	1.11E-05	12	12	12

1293	9.56E-04	9.779	9	12
1297	1.11E-05	12	12	12
1307	5.56E-05	10.8	9	12
1324	3.44E-04	9.839	9	12
1343	1.89E-04	9.706	9	12
1353	1.67E-04	10.2	9	12
1412	3.67E-04	10.121	9	12
1419	9.00E-04	9.679	9	12
1444	5.78E-04	9.846	9	12
1461	6.22E-04	9.661	9	12
1519	2.44E-04	9.682	9	12
1522	4.11E-04	9.676	9	12
1531	0.002	9.286	9	12
1548	0.001	9.263	9	12
1576	4.22E-04	9.658	9	12
1615	2.44E-04	10	9	12
1620	2.33E-04	10.095	9	12
1625	6.44E-04	9.948	9	12
1633	1.56E-04	9.5	9	12
1660	2.67E-04	9.833	9	12
1678	3.33E-04	9.9	9	12
1714	2.89E-04	9.654	9	12
1717	4.22E-04	9.974	9	12
1727	2.00E-04	10.333	9	12
1769	2.67E-04	10.25	9	12
1786	2.44E-04	10.182	9	12
1812	7.11E-04	9.484	9	12
1814	3.78E-04	10.324	9	12
1840	1.33E-04	10.333	9	12
1852	5.22E-04	9.809	9	12
1902	3.33E-04	9.8	9	12
1910	5.56E-04	9.74	9	12
1959	2.78E-04	10.12	9	12
1960	2.11E-04	9.947	9	12
1962	9.89E-04	9.865	9	12
1981	7.78E-05	10.143	9	12
2042	7.78E-05	10.571	9	12
3	7.00E-04	10.143	9	11
5	3.33E-05	10	9	11
6	7.78E-05	10.286	9	11
12	2.67E-04	10.333	9	11
14	3.22E-04	9.793	9	11
41	1.67E-04	9.733	9	11

43	1.11E-04	10	9	11
45	1.67E-04	10.067	9	11
53	1.22E-04	9.727	9	11
81	6.67E-05	10	9	11
83	2.00E-04	9.889	9	11
85	6.44E-04	9.397	9	11
119	1.11E-04	9.8	9	11
134	1.89E-04	9.647	9	11
174	3.67E-04	9.515	9	11
178	4.89E-04	9.977	9	11
180	2.67E-04	9.458	9	11
188	3.89E-04	9.486	9	11
220	1.78E-04	9.5	9	11
224	4.44E-05	9.75	9	11
232	1.33E-04	10.333	10	11
235	7.11E-04	9.641	9	11
244	6.22E-04	9.411	9	11
249	2.00E-04	9.667	9	11
261	1.33E-04	9.833	9	11
268	6.00E-04	9.204	9	11
274	0.002	9.314	9	11
281	1.00E-04	9.889	9	11
286	2.00E-04	9.889	9	11
291	4.89E-04	9.182	9	11
305	9.33E-04	9.202	9	11
306	8.00E-04	9.444	9	11
319	3.11E-04	9.464	9	11
331	9.78E-04	9.614	9	11
342	5.44E-04	9.531	9	11
357	1.44E-04	9.615	9	11
368	5.11E-04	9.261	9	11
391	5.56E-05	9.8	9	11
393	6.67E-05	10	9	11
396	4.00E-04	9.667	9	11
401	2.89E-04	9.346	9	11
405	3.33E-05	10	9	11
412	5.22E-04	9.255	9	11
456	8.89E-05	9.625	9	11
461	0.001	9.114	9	11
476	1.00E-04	10	9	11
478	8.89E-05	9.75	9	11
493	5.56E-04	9.72	9	11
497	0.001	9.258	9	11

508	6.67E-05	10.333	9	11
509	1.78E-04	10.25	10	11
517	1.56E-04	9.5	9	11
527	7.78E-05	9.857	9	11
541	2.44E-04	9.591	9	11
552	2.11E-04	10	9	11
586	6.67E-04	9.1	9	11
591	6.67E-05	9.667	9	11
594	0.007	9.355	9	11
595	1.00E-04	10	9	11
600	1.56E-04	9.929	9	11
603	0.001	9.117	9	11
623	3.33E-05	10	9	11
628	1.33E-04	9.5	9	11
633	8.89E-05	9.625	9	11
644	0.001	9.274	9	11
646	2.22E-04	9.95	9	11
661	1.78E-04	9.5	9	11
671	1.22E-04	9.636	9	11
712	1.11E-05	11	11	11
716	2.00E-04	9.333	9	11
733	4.67E-04	9.429	9	11
738	1.89E-04	9.529	9	11
750	7.78E-05	9.571	9	11
762	3.33E-04	9.933	9	11
787	1.11E-05	11	11	11
788	1.56E-04	9.429	9	11
803	0.001	9.461	9	11
806	1.11E-05	11	11	11
807	1.11E-04	9.7	9	11
818	0.001	9.42	9	11
841	0.001	9.246	9	11
842	2.22E-04	9.85	9	11
878	8.89E-05	10	9	11
882	5.33E-04	9.458	9	11
885	1.89E-04	10	9	11
909	4.44E-04	9.65	9	11
910	2.56E-04	9.522	9	11
935	2.11E-04	9.632	9	11
959	2.22E-04	9.6	9	11
974	2.44E-04	9.409	9	11
998	6.67E-05	9.833	9	11
1053	1.67E-04	9.467	9	11

1064	1.67E-04	9.4	9	11
1073	2.00E-04	9.5	9	11
1085	6.67E-05	9.667	9	11
1092	5.56E-05	9.8	9	11
1095	3.56E-04	9.625	9	11
1107	1.11E-05	11	11	11
1114	2.56E-04	9.783	9	11
1119	3.44E-04	9.419	9	11
1120	2.00E-04	9.722	9	11
1144	5.00E-04	9.689	9	11
1145	2.00E-04	9.611	9	11
1147	4.44E-05	10.5	10	11
1160	8.44E-04	9.447	9	11
1162	3.56E-04	9.562	9	11
1171	2.11E-04	9.632	9	11
1172	2.56E-04	9.435	9	11
1176	2.22E-05	10	9	11
1181	6.67E-05	9.667	9	11
1182	3.33E-04	9.5	9	11
1184	1.22E-04	9.364	9	11
1185	1.11E-05	11	11	11
1195	8.89E-05	10	9	11
1201	1.22E-04	9.364	9	11
1203	1.00E-04	9.444	9	11
1215	2.44E-04	9.5	9	11
1219	1.67E-04	9.6	9	11
1233	1.11E-05	11	11	11
1241	1.11E-05	11	11	11
1269	1.11E-05	11	11	11
1270	1.89E-04	9.588	9	11
1272	8.22E-04	9.27	9	11
1273	1.11E-05	11	11	11
1277	2.22E-04	9.85	9	11
1289	3.78E-04	9.294	9	11
1299	1.22E-04	9.545	9	11
1310	2.44E-04	9.818	9	11
1316	1.56E-04	9.714	9	11
1350	7.78E-05	9.714	9	11
1354	1.56E-04	9.714	9	11
1374	9.44E-04	9.471	9	11
1386	1.67E-04	9.667	9	11
1388	6.67E-05	9.833	9	11
1400	2.33E-04	9.476	9	11

1402	1.33E-04	9.583	9	11
1406	6.67E-05	9.667	9	11
1425	1.89E-04	9.941	9	11
1433	2.00E-04	9.722	9	11
1451	5.11E-04	9.435	9	11
1459	1.67E-04	9.933	9	11
1464	1.56E-04	9.857	9	11
1468	8.89E-05	9.75	9	11
1477	1.56E-04	9.714	9	11
1487	1.11E-04	9.7	9	11
1543	1.67E-04	9.533	9	11
1546	3.22E-04	9.655	9	11
1567	1.78E-04	9.75	9	11
1571	3.89E-04	9.543	9	11
1602	1.78E-04	10.062	9	11
1603	1.11E-04	9.4	9	11
1617	4.00E-04	9.667	9	11
1622	5.56E-05	9.4	9	11
1629	1.56E-04	9.643	9	11
1635	7.78E-05	9.857	9	11
1639	2.00E-04	9.556	9	11
1644	9.33E-04	9.119	9	11
1650	2.89E-04	9.231	9	11
1665	1.78E-04	9.625	9	11
1676	2.44E-04	9.545	9	11
1684	8.00E-04	9.653	9	11
1693	3.89E-04	9.429	9	11
1695	1.22E-04	9.636	9	11
1719	1.44E-04	9.462	9	11
1726	6.33E-04	9.754	9	11
1735	1.22E-04	9.455	9	11
1738	4.33E-04	9.359	9	11
1742	3.22E-04	9.483	9	11
1759	4.56E-04	9.61	9	11
1763	1.11E-04	9.6	9	11
1793	5.00E-04	9.422	9	11
1800	2.44E-04	9.682	9	11
1806	6.00E-04	9.352	9	11
1811	8.89E-05	9.75	9	11
1834	1.44E-04	9.462	9	11
1836	1.11E-04	9.6	9	11
1843	4.44E-05	9.75	9	11
1849	3.89E-04	9.343	9	11

1862	1.67E-04	9.733	9	11
1873	6.67E-05	10	9	11
1875	3.67E-04	9.424	9	11
1876	6.67E-05	10	9	11
1881	1.00E-04	9.667	9	11
1886	2.22E-04	9.7	9	11
1887	1.33E-04	9.667	9	11
1913	6.56E-04	9.407	9	11
1917	4.89E-04	9.818	9	11
1925	4.44E-05	10.5	10	11
1936	5.11E-04	9.174	9	11
1939	3.11E-04	9.679	9	11
1945	8.89E-05	9.625	9	11
1968	2.33E-04	10.048	9	11
1997	3.11E-04	9.536	9	11
2001	5.56E-05	9.8	9	11
2026	1.11E-05	11	11	11
2034	1.11E-05	11	11	11
2070	0.002	9.467	9	11
2071	7.11E-04	9.359	9	11
2073	1.67E-04	9.267	9	11
7	8.89E-05	10	10	10
10	2.67E-04	9.333	9	10
17	2.56E-04	9.348	9	10
20	1.11E-05	10	10	10
28	4.44E-05	9.5	9	10
30	5.33E-04	9.333	9	10
34	4.44E-05	9.25	9	10
35	0.002	9.018	9	10
51	6.67E-05	9.167	9	10
54	7.78E-05	9.143	9	10
56	1.11E-05	10	10	10
68	3.33E-04	9.1	9	10
72	1.11E-05	10	10	10
74	1.00E-04	9.556	9	10
75	1.11E-04	9.4	9	10
82	4.67E-04	9.238	9	10
88	2.44E-04	9.5	9	10
96	6.67E-05	9.333	9	10
99	2.22E-05	10	10	10
102	2.22E-05	9.5	9	10
107	2.22E-05	9.5	9	10
110	6.67E-05	9.5	9	10

117	1.11E-04	9.1	9	10
121	2.67E-04	9.167	9	10
124	3.33E-05	9.333	9	10
125	7.78E-05	9.429	9	10
129	2.22E-05	9.5	9	10
132	7.44E-04	9.119	9	10
139	5.56E-05	9.2	9	10
148	1.11E-04	9.3	9	10
157	1.11E-04	9.3	9	10
158	2.56E-04	9.565	9	10
165	3.33E-05	9.667	9	10
166	4.22E-04	9.474	9	10
168	1.11E-04	9.6	9	10
169	1.67E-04	9.467	9	10
171	9.78E-04	9.136	9	10
185	5.00E-04	9.156	9	10
187	1.33E-04	9.083	9	10
191	6.67E-05	9.5	9	10
196	4.44E-05	9.5	9	10
202	7.33E-04	9.136	9	10
205	1.44E-04	9.077	9	10
208	3.33E-05	9.333	9	10
209	1.22E-04	9.273	9	10
214	1.11E-04	9.1	9	10
215	1.44E-04	9.462	9	10
217	2.22E-04	9.3	9	10
221	2.00E-04	9.056	9	10
222	7.78E-05	9.286	9	10
223	1.78E-04	9.438	9	10
226	1.33E-04	9.417	9	10
228	1.44E-04	9.462	9	10
242	1.11E-05	10	10	10
248	1.44E-04	9.462	9	10
252	2.00E-04	9.389	9	10
258	6.67E-05	9.5	9	10
264	6.67E-05	9.5	9	10
265	1.00E-04	9.222	9	10
267	8.89E-05	9.375	9	10
301	9.56E-04	9.116	9	10
304	8.00E-04	9.222	9	10
318	5.78E-04	9.019	9	10
321	1.11E-04	9.3	9	10
323	1.11E-04	9.1	9	10

335	1.00E-04	9.444	9	10
348	6.67E-05	9.333	9	10
362	7.78E-05	9.286	9	10
370	4.44E-05	9.25	9	10
381	5.56E-05	9.6	9	10
386	1.33E-04	9.167	9	10
389	3.33E-05	9.333	9	10
392	1.00E-04	9.667	9	10
395	1.33E-04	9.333	9	10
398	3.33E-05	9.333	9	10
400	3.33E-05	9.333	9	10
404	5.56E-05	9.4	9	10
406	1.22E-04	9.545	9	10
416	2.22E-05	10	10	10
429	2.33E-04	9.381	9	10
435	1.11E-04	9.4	9	10
438	2.22E-04	9.6	9	10
442	1.78E-04	9.625	9	10
443	7.78E-05	9.714	9	10
444	2.22E-04	9.05	9	10
452	6.67E-05	9.833	9	10
453	8.89E-05	10	10	10
454	2.00E-04	9.556	9	10
459	1.11E-04	9.5	9	10
466	3.33E-05	9.667	9	10
468	1.44E-04	9.077	9	10
474	1.11E-04	9.2	9	10
480	2.22E-05	10	10	10
481	5.67E-04	9.196	9	10
483	2.22E-05	9.5	9	10
484	7.78E-05	9.429	9	10
490	1.11E-04	9.4	9	10
495	1.11E-04	9.4	9	10
501	1.11E-05	10	10	10
502	6.67E-05	9.333	9	10
505	2.89E-04	9.115	9	10
507	3.56E-04	9.188	9	10
510	5.56E-05	9.2	9	10
513	4.44E-05	9.5	9	10
514	4.00E-04	9.222	9	10
525	8.89E-05	9.75	9	10
529	1.11E-04	9.1	9	10
530	1.56E-04	9.214	9	10

532	2.44E-04	9.591	9	10
536	1.11E-04	9.1	9	10
545	1.11E-05	10	10	10
546	6.67E-05	9.5	9	10
550	1.56E-04	9.643	9	10
551	3.33E-05	9.333	9	10
558	8.67E-04	9.218	9	10
559	3.33E-05	9.333	9	10
561	3.33E-05	9.333	9	10
563	2.22E-05	9.5	9	10
564	3.33E-05	9.667	9	10
568	5.56E-05	9.4	9	10
569	5.56E-05	9.4	9	10
573	7.78E-05	9.143	9	10
576	1.44E-04	9.615	9	10
578	1.11E-04	9.4	9	10
579	7.78E-05	9.143	9	10
588	1.78E-04	9.25	9	10
590	5.56E-05	9.6	9	10
593	1.22E-04	9.545	9	10
599	4.44E-05	9.5	9	10
608	1.11E-05	10	10	10
613	1.11E-04	9.6	9	10
619	1.11E-05	10	10	10
629	1.11E-04	9.3	9	10
634	1.56E-04	9.143	9	10
635	2.89E-04	9.231	9	10
640	4.78E-04	9.07	9	10
648	1.56E-04	9.714	9	10
651	6.33E-04	9.018	9	10
652	1.11E-04	9.3	9	10
664	1.56E-04	9.357	9	10
667	2.00E-04	9.278	9	10
668	8.89E-05	9.5	9	10
673	1.22E-04	9.091	9	10
678	1.56E-04	9.143	9	10
688	1.33E-04	9.333	9	10
691	5.22E-04	9.149	9	10
707	2.22E-05	9.5	9	10
708	1.00E-04	9.222	9	10
711	3.33E-05	9.333	9	10
720	1.44E-04	9.077	9	10
732	4.67E-04	9.143	9	10

734	8.89E-05	9.125	9	10
739	3.33E-05	9.333	9	10
745	6.67E-05	9.167	9	10
746	1.11E-05	10	10	10
747	3.33E-05	9.333	9	10
748	1.11E-04	9.2	9	10
759	4.44E-05	9.25	9	10
767	2.78E-04	9.08	9	10
775	4.44E-05	9.25	9	10
778	3.33E-05	9.333	9	10
781	1.89E-04	9.118	9	10
782	1.11E-05	10	10	10
786	6.56E-04	9.22	9	10
789	3.11E-04	9.036	9	10
812	3.78E-04	9.294	9	10
814	1.56E-04	9.071	9	10
816	1.11E-05	10	10	10
826	1.11E-05	10	10	10
828	1.11E-05	10	10	10
837	1.11E-05	10	10	10
843	2.67E-04	9.042	9	10
844	1.11E-05	10	10	10
848	6.67E-05	9.333	9	10
860	8.89E-05	9.125	9	10
872	2.78E-04	9.08	9	10
876	2.89E-04	9.077	9	10
921	1.11E-05	10	10	10
940	1.11E-05	10	10	10
941	1.11E-05	10	10	10
943	1.11E-05	10	10	10
957	1.33E-04	9.5	9	10
966	1.11E-05	10	10	10
989	1.11E-05	10	10	10
994	6.67E-05	9.167	9	10
1001	8.89E-05	9.625	9	10
1005	2.22E-05	9.5	9	10
1006	4.22E-04	9.184	9	10
1013	1.11E-05	10	10	10
1014	1.11E-05	10	10	10
1016	2.00E-04	9.556	9	10
1028	2.00E-04	9.167	9	10
1037	6.67E-05	9.5	9	10
1040	5.56E-05	9.2	9	10

1042	1.11E-05	10	10	10
1043	1.22E-04	9.636	9	10
1044	1.22E-04	9.364	9	10
1049	2.22E-05	9.5	9	10
1056	4.44E-05	9.5	9	10
1059	1.11E-05	10	10	10
1061	5.56E-05	9.2	9	10
1075	4.44E-05	9.5	9	10
1077	6.67E-05	9.167	9	10
1080	1.11E-04	9.1	9	10
1097	1.11E-04	9.3	9	10
1098	3.33E-05	9.333	9	10
1104	5.89E-04	9.34	9	10
1110	6.67E-05	9.167	9	10
1111	8.89E-05	9.75	9	10
1112	1.11E-04	9.1	9	10
1128	4.44E-05	9.5	9	10
1134	1.89E-04	9.412	9	10
1138	1.11E-05	10	10	10
1157	7.78E-05	9.143	9	10
1159	1.22E-04	9.273	9	10
1163	2.22E-04	9.1	9	10
1164	1.22E-04	9.545	9	10
1177	6.67E-05	9.167	9	10
1183	3.89E-04	9.371	9	10
1188	1.11E-05	10	10	10
1191	8.89E-05	9.25	9	10
1206	1.22E-04	9.182	9	10
1209	5.78E-04	9.077	9	10
1211	2.22E-05	9.5	9	10
1222	2.22E-05	9.5	9	10
1223	1.89E-04	9.294	9	10
1232	6.22E-04	9.036	9	10
1235	2.89E-04	9.231	9	10
1237	3.33E-05	9.333	9	10
1240	2.22E-05	9.5	9	10
1244	8.89E-05	9.125	9	10
1246	2.89E-04	9.154	9	10
1252	1.22E-04	9.545	9	10
1253	1.78E-04	9.062	9	10
1255	2.22E-04	9.05	9	10
1256	4.44E-05	9.25	9	10
1261	2.78E-04	9.04	9	10

1268	1.22E-04	9.091	9	10
1271	7.78E-05	9.143	9	10
1278	1.33E-04	9.417	9	10
1291	6.67E-05	9.333	9	10
1301	6.67E-05	9.333	9	10
1302	6.67E-05	9.167	9	10
1308	4.44E-05	9.5	9	10
1320	3.00E-04	9.037	9	10
1330	2.11E-04	9.474	9	10
1333	8.89E-05	9.125	9	10
1338	5.56E-05	9.4	9	10
1339	1.11E-04	9.6	9	10
1340	4.44E-05	9.5	9	10
1341	4.44E-05	9.5	9	10
1362	1.11E-04	9.1	9	10
1368	3.33E-05	9.333	9	10
1369	6.67E-05	9.667	9	10
1377	6.67E-05	9.333	9	10
1378	3.33E-05	9.667	9	10
1379	3.33E-05	9.667	9	10
1381	4.44E-05	9.5	9	10
1385	1.22E-04	9.545	9	10
1399	5.56E-05	9.2	9	10
1409	1.11E-05	10	10	10
1410	7.78E-05	9.286	9	10
1411	3.33E-05	9.333	9	10
1418	1.56E-04	9.214	9	10
1420	7.78E-05	9.143	9	10
1421	3.33E-05	9.333	9	10
1423	1.00E-04	9.333	9	10
1432	1.11E-04	9.2	9	10
1435	1.22E-04	9.091	9	10
1440	1.11E-04	9.3	9	10
1445	1.00E-04	9.556	9	10
1447	8.89E-05	9.25	9	10
1448	4.44E-05	9.5	9	10
1454	1.11E-05	10	10	10
1456	1.56E-04	9.071	9	10
1462	2.22E-05	10	10	10
1467	8.89E-05	9.25	9	10
1469	1.11E-05	10	10	10
1475	3.33E-05	9.333	9	10
1481	4.44E-05	9.25	9	10

1493	1.00E-04	9.444	9	10
1495	8.89E-05	9.25	9	10
1503	1.11E-04	9.1	9	10
1504	8.89E-05	9.75	9	10
1521	1.33E-04	9.333	9	10
1528	4.44E-05	9.25	9	10
1532	6.67E-05	9.167	9	10
1533	7.78E-05	9.286	9	10
1536	6.67E-05	9.167	9	10
1539	3.33E-05	9.333	9	10
1551	1.11E-04	9.5	9	10
1552	6.67E-05	9.333	9	10
1553	3.33E-04	9.1	9	10
1554	6.67E-05	9.667	9	10
1556	6.67E-05	9.333	9	10
1564	7.11E-04	9.297	9	10
1572	1.22E-04	9.091	9	10
1573	2.89E-04	9.577	9	10
1578	1.11E-04	9.4	9	10
1584	1.22E-04	9.273	9	10
1585	2.22E-05	9.5	9	10
1589	6.67E-05	9.333	9	10
1593	1.33E-04	9.417	9	10
1594	7.78E-05	9.429	9	10
1599	3.33E-05	9.333	9	10
1605	1.33E-04	9.25	9	10
1609	1.89E-04	9.294	9	10
1616	8.89E-05	9.25	9	10
1621	1.56E-04	9.5	9	10
1627	8.89E-05	9.125	9	10
1637	1.33E-04	9.333	9	10
1646	1.22E-04	9.273	9	10
1652	1.00E-04	9.444	9	10
1653	1.33E-04	9.25	9	10
1654	7.78E-05	9.429	9	10
1661	1.78E-04	9.125	9	10
1666	6.67E-05	9.167	9	10
1669	6.67E-05	9.167	9	10
1675	7.78E-05	9.286	9	10
1689	1.33E-04	9.5	9	10
1703	2.22E-04	9.5	9	10
1704	8.89E-05	9.375	9	10
1713	5.33E-04	9.667	9	10

1715	6.67E-05	9.167	9	10
1718	2.44E-04	9.273	9	10
1725	2.22E-05	9.5	9	10
1728	1.22E-04	9.182	9	10
1730	2.22E-05	9.5	9	10
1734	8.89E-05	9.375	9	10
1744	6.67E-05	9.333	9	10
1747	3.00E-04	9.185	9	10
1758	1.00E-04	9.444	9	10
1768	1.00E-04	9.556	9	10
1770	2.78E-04	9.28	9	10
1774	3.33E-04	9.333	9	10
1775	4.44E-05	9.5	9	10
1783	8.89E-05	9.5	9	10
1789	8.89E-05	10	10	10
1795	6.67E-05	9.167	9	10
1801	3.00E-04	9.296	9	10
1802	1.56E-04	9.071	9	10
1803	1.11E-05	10	10	10
1807	1.56E-04	9.571	9	10
1808	8.89E-05	9.25	9	10
1818	1.00E-04	9.222	9	10
1820	2.22E-05	10	10	10
1827	1.33E-04	9.333	9	10
1830	1.22E-04	9.273	9	10
1839	1.56E-04	9.214	9	10
1848	1.44E-04	9.692	9	10
1855	6.67E-05	9.5	9	10
1864	1.56E-04	9.143	9	10
1883	5.56E-05	9.2	9	10
1891	1.11E-04	9.6	9	10
1894	1.56E-04	9.143	9	10
1896	1.11E-05	10	10	10
1916	6.67E-05	9.333	9	10
1918	2.11E-04	9.105	9	10
1919	4.44E-05	9.75	9	10
1921	2.22E-04	9.3	9	10
1922	7.78E-05	9.143	9	10
1924	1.78E-04	9.25	9	10
1928	1.11E-04	9.5	9	10
1929	5.56E-05	9.2	9	10
1931	2.22E-05	9.5	9	10
1935	3.56E-04	9.062	9	10

1951	7.78E-05	9.286	9	10
1958	6.67E-05	9.5	9	10
1961	1.11E-04	9.2	9	10
1974	1.11E-04	9.4	9	10
1975	1.78E-04	9.125	9	10
1977	1.33E-04	9.417	9	10
1984	5.56E-05	9.2	9	10
1990	3.33E-05	9.333	9	10
1992	8.89E-05	9.25	9	10
1994	1.33E-04	9.333	9	10
2000	3.33E-05	9.333	9	10
2013	3.33E-05	9.667	9	10
2024	8.89E-05	9.75	9	10
2031	1.11E-04	9.4	9	10
2037	4.44E-05	9.25	9	10
2040	1.33E-04	9.333	9	10
2046	8.89E-05	9.25	9	10
2048	4.44E-05	9.25	9	10
2053	1.78E-04	9.25	9	10
2055	1.33E-04	9.5	9	10
2066	5.56E-05	9.2	9	10
2072	6.67E-05	9.5	9	10
2	1.78E-04	9	9	9
4	1.11E-05	9	9	9
8	1.11E-05	9	9	9
9	1.11E-05	9	9	9
11	8.89E-05	9	9	9
13	1.78E-04	9	9	9
15	8.89E-05	9	9	9
16	3.33E-05	9	9	9
18	8.89E-05	9	9	9
19	2.22E-05	9	9	9
21	1.11E-05	9	9	9
22	1.11E-05	9	9	9
24	8.89E-05	9	9	9
25	1.11E-05	9	9	9
26	3.33E-05	9	9	9
27	1.11E-05	9	9	9
29	1.22E-04	9	9	9
31	1.11E-05	9	9	9
32	6.67E-05	9	9	9
33	2.22E-05	9	9	9
36	2.22E-05	9	9	9

37	1.11E-05	9	9	9
38	1.11E-05	9	9	9
39	8.89E-05	9	9	9
40	1.22E-04	9	9	9
44	2.67E-04	9	9	9
46	1.11E-05	9	9	9
48	2.67E-04	9	9	9
50	8.89E-05	9	9	9
52	2.22E-05	9	9	9
55	5.56E-05	9	9	9
57	2.11E-04	9	9	9
59	1.78E-04	9	9	9
60	1.67E-04	9	9	9
62	1.44E-04	9	9	9
64	5.56E-05	9	9	9
65	2.22E-05	9	9	9
66	1.11E-05	9	9	9
67	3.33E-05	9	9	9
69	1.11E-05	9	9	9
71	1.11E-05	9	9	9
73	1.11E-05	9	9	9
78	1.11E-05	9	9	9
79	1.11E-05	9	9	9
80	1.11E-05	9	9	9
84	1.11E-05	9	9	9
86	3.00E-04	9	9	9
87	2.22E-05	9	9	9
89	3.33E-05	9	9	9
90	3.33E-05	9	9	9
91	1.11E-05	9	9	9
92	3.33E-05	9	9	9
93	3.33E-05	9	9	9
94	6.67E-05	9	9	9
95	3.33E-05	9	9	9
97	2.22E-04	9	9	9
98	1.11E-05	9	9	9
101	3.33E-05	9	9	9
103	3.33E-05	9	9	9
104	3.33E-05	9	9	9
105	4.44E-05	9	9	9
109	8.89E-05	9	9	9
111	2.22E-05	9	9	9
112	4.11E-04	9	9	9

113	1.44E-04	9	9	9
114	2.67E-04	9	9	9
115	2.67E-04	9	9	9
116	2.22E-05	9	9	9
118	2.22E-05	9	9	9
120	2.22E-05	9	9	9
122	3.33E-05	9	9	9
123	1.11E-05	9	9	9
126	3.33E-05	9	9	9
127	3.33E-05	9	9	9
131	1.11E-05	9	9	9
133	2.67E-04	9	9	9
135	2.22E-05	9	9	9
136	2.22E-05	9	9	9
137	1.56E-04	9	9	9
138	1.78E-04	9	9	9
140	1.11E-05	9	9	9
142	2.22E-05	9	9	9
143	2.22E-05	9	9	9
144	3.33E-05	9	9	9
145	1.11E-05	9	9	9
146	1.11E-05	9	9	9
147	1.11E-05	9	9	9
149	3.56E-04	9	9	9
150	8.89E-05	9	9	9
151	8.89E-05	9	9	9
152	8.89E-05	9	9	9
153	8.89E-05	9	9	9
154	1.11E-05	9	9	9
156	2.22E-05	9	9	9
160	1.11E-05	9	9	9
162	2.22E-04	9	9	9
164	4.44E-05	9	9	9
170	2.22E-05	9	9	9
172	3.33E-05	9	9	9
173	3.33E-05	9	9	9
176	2.22E-05	9	9	9
177	1.11E-05	9	9	9
179	1.11E-05	9	9	9
181	2.22E-05	9	9	9
182	3.33E-05	9	9	9
183	2.11E-04	9	9	9
184	2.22E-05	9	9	9

189	2.22E-05	9	9	9
190	2.22E-05	9	9	9
192	1.78E-04	9	9	9
194	2.22E-05	9	9	9
195	8.89E-05	9	9	9
203	1.11E-05	9	9	9
204	8.89E-05	9	9	9
206	3.33E-05	9	9	9
207	3.33E-05	9	9	9
210	1.78E-04	9	9	9
211	8.89E-05	9	9	9
212	3.33E-05	9	9	9
213	2.22E-05	9	9	9
216	1.11E-05	9	9	9
218	1.11E-05	9	9	9
225	1.11E-05	9	9	9
227	5.56E-05	9	9	9
229	2.67E-04	9	9	9
230	5.56E-05	9	9	9
231	5.56E-05	9	9	9
233	1.11E-04	9	9	9
234	3.33E-05	9	9	9
236	4.44E-04	9	9	9
237	5.56E-05	9	9	9
238	3.33E-05	9	9	9
239	5.56E-05	9	9	9
240	2.22E-05	9	9	9
241	1.11E-05	9	9	9
243	2.67E-04	9	9	9
246	2.67E-04	9	9	9
250	1.11E-05	9	9	9
251	3.33E-05	9	9	9
253	1.11E-05	9	9	9
254	2.22E-05	9	9	9
255	5.33E-04	9	9	9
256	4.44E-05	9	9	9
257	3.33E-05	9	9	9
259	1.11E-05	9	9	9
262	8.89E-05	9	9	9
263	4.44E-05	9	9	9
266	1.11E-05	9	9	9
269	1.11E-05	9	9	9
270	1.11E-05	9	9	9

271	1.11E-05	9	9	9
272	1.78E-04	9	9	9
273	4.44E-05	9	9	9
275	5.56E-05	9	9	9
276	1.11E-05	9	9	9
277	7.78E-05	9	9	9
278	5.56E-05	9	9	9
279	7.78E-05	9	9	9
283	7.11E-04	9	9	9
284	3.56E-04	9	9	9
285	2.22E-05	9	9	9
290	2.67E-04	9	9	9
292	2.22E-05	9	9	9
293	5.67E-04	9	9	9
294	7.78E-05	9	9	9
296	1.56E-04	9	9	9
297	3.33E-04	9	9	9
298	5.33E-04	9	9	9
299	1.11E-05	9	9	9
300	3.33E-05	9	9	9
302	1.11E-05	9	9	9
303	2.67E-04	9	9	9
307	2.67E-04	9	9	9
309	2.22E-05	9	9	9
312	1.11E-05	9	9	9
313	1.11E-05	9	9	9
314	1.44E-04	9	9	9
315	2.67E-04	9	9	9
316	1.11E-05	9	9	9
317	3.33E-05	9	9	9
320	5.56E-04	9	9	9
322	3.00E-04	9	9	9
324	3.33E-05	9	9	9
325	3.56E-04	9	9	9
326	1.78E-04	9	9	9
327	3.78E-04	9	9	9
328	8.89E-05	9	9	9
329	1.11E-05	9	9	9
330	3.33E-05	9	9	9
333	3.33E-05	9	9	9
334	1.11E-05	9	9	9
336	1.44E-04	9	9	9
337	2.22E-05	9	9	9

339	1.11E-05	9	9	9
340	1.11E-05	9	9	9
341	1.11E-05	9	9	9
344	1.44E-04	9	9	9
346	1.11E-05	9	9	9
347	2.22E-05	9	9	9
350	6.67E-05	9	9	9
351	1.44E-04	9	9	9
352	1.11E-05	9	9	9
353	1.11E-05	9	9	9
354	1.11E-05	9	9	9
355	8.89E-05	9	9	9
356	4.44E-05	9	9	9
358	1.22E-04	9	9	9
359	3.56E-04	9	9	9
360	1.78E-04	9	9	9
361	2.33E-04	9	9	9
364	2.22E-05	9	9	9
365	3.33E-05	9	9	9
366	3.56E-04	9	9	9
367	3.33E-05	9	9	9
369	1.11E-04	9	9	9
371	3.33E-05	9	9	9
373	4.44E-05	9	9	9
374	5.56E-05	9	9	9
375	2.22E-05	9	9	9
376	2.22E-05	9	9	9
377	2.22E-05	9	9	9
378	3.33E-05	9	9	9
380	3.33E-05	9	9	9
382	4.44E-05	9	9	9
383	2.22E-05	9	9	9
384	2.22E-05	9	9	9
385	1.11E-05	9	9	9
387	1.11E-05	9	9	9
388	3.33E-05	9	9	9
394	2.22E-05	9	9	9
397	3.11E-04	9	9	9
399	1.11E-05	9	9	9
402	2.22E-05	9	9	9
409	2.22E-05	9	9	9
410	1.11E-05	9	9	9
413	1.78E-04	9	9	9

414	4.44E-05	9	9	9
415	3.33E-05	9	9	9
417	8.00E-04	9	9	9
418	4.44E-04	9	9	9
419	8.89E-05	9	9	9
420	8.89E-05	9	9	9
421	3.33E-05	9	9	9
422	4.44E-05	9	9	9
424	4.44E-05	9	9	9
425	3.33E-05	9	9	9
427	1.11E-05	9	9	9
428	1.56E-04	9	9	9
430	1.11E-05	9	9	9
431	6.67E-05	9	9	9
434	2.22E-05	9	9	9
437	7.11E-04	9	9	9
439	3.33E-05	9	9	9
441	5.56E-05	9	9	9
445	3.33E-05	9	9	9
446	2.89E-04	9	9	9
447	2.22E-05	9	9	9
448	4.44E-05	9	9	9
449	2.22E-05	9	9	9
450	1.78E-04	9	9	9
455	2.22E-05	9	9	9
457	4.44E-05	9	9	9
460	2.22E-05	9	9	9
462	2.22E-05	9	9	9
463	2.22E-05	9	9	9
464	6.67E-05	9	9	9
465	1.11E-05	9	9	9
467	3.33E-05	9	9	9
469	7.78E-05	9	9	9
471	8.89E-05	9	9	9
472	4.44E-05	9	9	9
477	1.11E-05	9	9	9
479	3.33E-05	9	9	9
482	1.11E-05	9	9	9
486	4.44E-05	9	9	9
487	4.44E-05	9	9	9
488	1.11E-05	9	9	9
489	1.11E-05	9	9	9
491	2.22E-05	9	9	9

494	4.44E-05	9	9	9
496	2.22E-05	9	9	9
498	2.22E-05	9	9	9
499	1.67E-04	9	9	9
500	1.11E-05	9	9	9
504	5.33E-04	9	9	9
506	1.78E-04	9	9	9
512	4.44E-05	9	9	9
515	2.22E-05	9	9	9
516	1.11E-05	9	9	9
518	1.78E-04	9	9	9
520	3.56E-04	9	9	9
521	2.22E-05	9	9	9
522	3.33E-05	9	9	9
523	6.67E-04	9	9	9
524	1.00E-04	9	9	9
526	4.44E-05	9	9	9
531	1.11E-05	9	9	9
534	1.11E-05	9	9	9
535	1.11E-05	9	9	9
540	1.11E-05	9	9	9
542	1.11E-05	9	9	9
543	4.44E-05	9	9	9
544	1.11E-05	9	9	9
547	2.22E-04	9	9	9
548	1.11E-05	9	9	9
549	1.44E-04	9	9	9
556	2.67E-04	9	9	9
560	2.22E-05	9	9	9
562	2.22E-05	9	9	9
565	2.22E-05	9	9	9
567	5.56E-05	9	9	9
570	1.11E-05	9	9	9
571	8.89E-05	9	9	9
572	1.11E-05	9	9	9
574	3.33E-05	9	9	9
577	7.78E-05	9	9	9
580	1.11E-05	9	9	9
581	1.11E-05	9	9	9
582	3.33E-05	9	9	9
583	1.11E-05	9	9	9
585	2.22E-05	9	9	9
587	2.22E-05	9	9	9

592	1.11E-05	9	9	9
596	3.33E-05	9	9	9
598	3.33E-05	9	9	9
601	1.11E-05	9	9	9
602	3.33E-05	9	9	9
604	3.33E-05	9	9	9
605	1.11E-05	9	9	9
606	2.22E-04	9	9	9
607	4.44E-05	9	9	9
609	1.11E-05	9	9	9
614	1.11E-05	9	9	9
615	7.78E-05	9	9	9
616	5.56E-05	9	9	9
618	4.44E-05	9	9	9
620	1.11E-05	9	9	9
621	4.44E-05	9	9	9
622	1.11E-05	9	9	9
624	1.11E-05	9	9	9
625	1.78E-04	9	9	9
626	1.78E-04	9	9	9
627	1.11E-05	9	9	9
631	5.56E-05	9	9	9
632	1.67E-04	9	9	9
636	1.11E-05	9	9	9
637	5.56E-05	9	9	9
638	1.11E-05	9	9	9
639	5.56E-05	9	9	9
642	2.67E-04	9	9	9
643	3.33E-05	9	9	9
645	2.22E-05	9	9	9
647	1.11E-05	9	9	9
649	1.11E-04	9	9	9
650	2.22E-05	9	9	9
653	2.22E-05	9	9	9
655	1.11E-05	9	9	9
656	3.33E-05	9	9	9
657	1.11E-05	9	9	9
658	2.22E-05	9	9	9
659	4.44E-05	9	9	9
660	1.11E-05	9	9	9
662	1.11E-05	9	9	9
663	1.44E-04	9	9	9
665	3.89E-04	9	9	9

666	1.11E-05	9	9	9
674	4.44E-05	9	9	9
675	1.11E-05	9	9	9
676	1.11E-05	9	9	9
677	1.11E-05	9	9	9
679	1.11E-05	9	9	9
680	6.67E-05	9	9	9
682	1.33E-04	9	9	9
685	4.44E-05	9	9	9
687	2.22E-05	9	9	9
689	2.22E-05	9	9	9
690	7.78E-05	9	9	9
692	1.11E-05	9	9	9
693	4.44E-05	9	9	9
694	1.11E-05	9	9	9
695	1.11E-05	9	9	9
696	1.11E-05	9	9	9
697	1.11E-05	9	9	9
699	2.22E-05	9	9	9
700	2.22E-05	9	9	9
701	2.22E-05	9	9	9
703	1.11E-05	9	9	9
706	2.22E-05	9	9	9
709	1.11E-05	9	9	9
710	4.44E-05	9	9	9
713	2.67E-04	9	9	9
714	4.44E-05	9	9	9
717	1.11E-05	9	9	9
718	1.11E-05	9	9	9
719	7.78E-05	9	9	9
721	3.33E-05	9	9	9
722	2.22E-05	9	9	9
725	1.11E-05	9	9	9
726	1.11E-05	9	9	9
727	3.33E-05	9	9	9
728	1.11E-05	9	9	9
729	1.11E-04	9	9	9
730	2.22E-05	9	9	9
731	2.22E-05	9	9	9
735	2.22E-05	9	9	9
736	1.11E-05	9	9	9
741	1.11E-05	9	9	9
742	2.22E-05	9	9	9

744	1.33E-04	9	9	9
749	2.22E-05	9	9	9
751	1.11E-05	9	9	9
753	1.11E-05	9	9	9
754	3.33E-05	9	9	9
755	1.11E-05	9	9	9
756	8.89E-05	9	9	9
757	1.11E-05	9	9	9
758	1.11E-05	9	9	9
760	1.78E-04	9	9	9
763	1.11E-05	9	9	9
765	6.67E-05	9	9	9
766	1.11E-05	9	9	9
768	2.22E-05	9	9	9
769	2.22E-05	9	9	9
770	2.44E-04	9	9	9
771	3.33E-05	9	9	9
773	1.56E-04	9	9	9
774	8.89E-05	9	9	9
776	2.22E-05	9	9	9
777	1.11E-05	9	9	9
779	3.56E-04	9	9	9
780	2.67E-04	9	9	9
783	2.22E-05	9	9	9
785	1.78E-04	9	9	9
791	2.22E-05	9	9	9
792	2.22E-05	9	9	9
794	2.22E-05	9	9	9
795	1.11E-05	9	9	9
796	1.11E-05	9	9	9
799	1.11E-05	9	9	9
801	1.11E-05	9	9	9
804	8.89E-05	9	9	9
805	2.22E-05	9	9	9
810	8.89E-05	9	9	9
811	1.11E-05	9	9	9
819	4.44E-05	9	9	9
820	1.11E-05	9	9	9
823	1.11E-05	9	9	9
824	7.78E-05	9	9	9
825	1.11E-05	9	9	9
827	1.11E-05	9	9	9
832	2.22E-05	9	9	9

833	1.11E-05	9	9	9
834	2.22E-05	9	9	9
838	1.11E-05	9	9	9
839	6.67E-05	9	9	9
840	1.00E-04	9	9	9
845	2.22E-05	9	9	9
847	2.22E-05	9	9	9
852	1.11E-05	9	9	9
853	1.11E-05	9	9	9
854	3.33E-05	9	9	9
858	1.11E-05	9	9	9
859	3.33E-05	9	9	9
861	2.22E-05	9	9	9
862	1.11E-05	9	9	9
864	5.56E-05	9	9	9
866	6.67E-05	9	9	9
867	2.22E-05	9	9	9
868	1.11E-05	9	9	9
869	1.11E-05	9	9	9
871	2.22E-05	9	9	9
875	2.67E-04	9	9	9
877	1.11E-05	9	9	9
880	1.11E-05	9	9	9
881	3.33E-05	9	9	9
886	2.22E-05	9	9	9
888	1.11E-05	9	9	9
889	1.11E-05	9	9	9
893	1.11E-05	9	9	9
894	4.44E-05	9	9	9
896	2.22E-05	9	9	9
898	6.67E-05	9	9	9
899	2.22E-05	9	9	9
900	1.11E-05	9	9	9
901	6.67E-05	9	9	9
904	1.11E-05	9	9	9
905	2.22E-05	9	9	9
907	3.56E-04	9	9	9
908	2.22E-05	9	9	9
912	1.00E-04	9	9	9
914	1.33E-04	9	9	9
915	1.11E-05	9	9	9
916	1.11E-05	9	9	9
917	8.89E-05	9	9	9

919	3.33E-05	9	9	9
920	4.44E-05	9	9	9
922	2.22E-04	9	9	9
924	4.44E-05	9	9	9
925	2.22E-05	9	9	9
927	1.11E-05	9	9	9
928	2.22E-05	9	9	9
929	2.22E-05	9	9	9
931	3.33E-05	9	9	9
932	1.11E-05	9	9	9
934	3.33E-05	9	9	9
936	1.11E-05	9	9	9
938	1.11E-05	9	9	9
939	1.11E-05	9	9	9
942	1.11E-05	9	9	9
944	1.11E-05	9	9	9
945	2.67E-04	9	9	9
946	4.44E-05	9	9	9
947	3.56E-04	9	9	9
948	2.67E-04	9	9	9
949	1.11E-05	9	9	9
950	3.33E-05	9	9	9
952	2.22E-05	9	9	9
958	4.44E-05	9	9	9
960	1.11E-05	9	9	9
961	3.33E-05	9	9	9
962	4.44E-05	9	9	9
964	1.11E-05	9	9	9
967	1.11E-05	9	9	9
968	1.11E-05	9	9	9
969	1.11E-05	9	9	9
970	1.11E-05	9	9	9
971	1.11E-05	9	9	9
973	1.11E-05	9	9	9
977	1.11E-05	9	9	9
978	1.11E-05	9	9	9
980	5.56E-05	9	9	9
982	1.11E-05	9	9	9
983	2.22E-05	9	9	9
985	3.33E-05	9	9	9
987	1.11E-05	9	9	9
990	1.11E-05	9	9	9
992	1.11E-05	9	9	9

993	2.67E-04	9	9	9
995	1.11E-05	9	9	9
996	1.11E-05	9	9	9
997	1.11E-05	9	9	9
1002	2.22E-05	9	9	9
1003	1.11E-05	9	9	9
1004	2.22E-05	9	9	9
1007	4.44E-05	9	9	9
1008	1.11E-05	9	9	9
1011	1.11E-05	9	9	9
1012	1.11E-05	9	9	9
1015	1.11E-05	9	9	9
1017	5.56E-05	9	9	9
1018	5.33E-04	9	9	9
1019	1.11E-05	9	9	9
1020	3.33E-05	9	9	9
1021	1.11E-05	9	9	9
1022	1.11E-05	9	9	9
1023	6.67E-05	9	9	9
1024	3.33E-05	9	9	9
1025	3.33E-05	9	9	9
1026	1.11E-05	9	9	9
1030	3.33E-05	9	9	9
1031	1.11E-05	9	9	9
1032	1.11E-05	9	9	9
1034	8.89E-05	9	9	9
1035	2.67E-04	9	9	9
1036	3.56E-04	9	9	9
1038	1.11E-05	9	9	9
1041	1.22E-04	9	9	9
1045	1.11E-05	9	9	9
1046	1.44E-04	9	9	9
1047	1.11E-05	9	9	9
1048	2.22E-05	9	9	9
1050	3.33E-05	9	9	9
1051	2.22E-05	9	9	9
1052	1.11E-05	9	9	9
1054	1.11E-05	9	9	9
1055	8.89E-05	9	9	9
1057	2.22E-05	9	9	9
1060	2.22E-05	9	9	9
1062	4.44E-05	9	9	9
1063	4.44E-05	9	9	9

1065	1.11E-05	9	9	9
1066	3.33E-05	9	9	9
1067	1.11E-05	9	9	9
1068	1.11E-05	9	9	9
1069	6.67E-05	9	9	9
1070	6.67E-05	9	9	9
1071	8.89E-05	9	9	9
1072	3.33E-05	9	9	9
1074	4.44E-05	9	9	9
1076	2.22E-05	9	9	9
1078	1.11E-05	9	9	9
1079	3.56E-04	9	9	9
1081	1.11E-05	9	9	9
1082	7.78E-05	9	9	9
1083	2.22E-05	9	9	9
1084	8.89E-05	9	9	9
1086	2.22E-05	9	9	9
1087	1.11E-05	9	9	9
1088	7.78E-05	9	9	9
1089	5.56E-05	9	9	9
1090	3.33E-05	9	9	9
1091	1.11E-04	9	9	9
1093	8.89E-05	9	9	9
1094	5.56E-05	9	9	9
1096	3.89E-04	9	9	9
1102	4.44E-05	9	9	9
1103	2.22E-05	9	9	9
1105	4.44E-05	9	9	9
1106	2.22E-05	9	9	9
1108	2.22E-05	9	9	9
1109	5.56E-05	9	9	9
1113	5.56E-05	9	9	9
1115	6.67E-05	9	9	9
1117	3.33E-05	9	9	9
1118	1.11E-05	9	9	9
1121	2.22E-05	9	9	9
1122	5.56E-05	9	9	9
1123	1.33E-04	9	9	9
1124	2.22E-05	9	9	9
1125	1.11E-05	9	9	9
1126	4.44E-05	9	9	9
1127	1.11E-05	9	9	9
1129	4.44E-05	9	9	9

1130	1.11E-05	9	9	9
1131	1.33E-04	9	9	9
1132	5.56E-05	9	9	9
1136	3.33E-05	9	9	9
1139	1.11E-05	9	9	9
1140	1.11E-05	9	9	9
1141	4.44E-05	9	9	9
1142	1.11E-05	9	9	9
1143	1.11E-05	9	9	9
1146	2.22E-05	9	9	9
1149	5.56E-05	9	9	9
1150	1.11E-05	9	9	9
1151	5.56E-05	9	9	9
1152	1.11E-05	9	9	9
1153	2.22E-05	9	9	9
1154	2.22E-05	9	9	9
1155	2.22E-05	9	9	9
1158	2.22E-05	9	9	9
1165	4.44E-05	9	9	9
1166	8.89E-05	9	9	9
1167	2.22E-05	9	9	9
1168	2.22E-05	9	9	9
1169	2.22E-05	9	9	9
1170	1.11E-05	9	9	9
1173	2.22E-05	9	9	9
1174	2.22E-05	9	9	9
1175	1.11E-05	9	9	9
1178	1.11E-05	9	9	9
1179	1.00E-04	9	9	9
1180	1.11E-05	9	9	9
1189	8.89E-05	9	9	9
1190	2.22E-05	9	9	9
1192	1.11E-05	9	9	9
1193	1.11E-05	9	9	9
1194	1.11E-05	9	9	9
1196	1.11E-05	9	9	9
1199	4.44E-05	9	9	9
1200	1.11E-05	9	9	9
1204	4.44E-05	9	9	9
1205	1.11E-05	9	9	9
1207	1.11E-05	9	9	9
1208	4.44E-05	9	9	9
1212	1.11E-05	9	9	9

1213	5.56E-05	9	9	9
1224	1.11E-05	9	9	9
1225	5.56E-05	9	9	9
1226	1.78E-04	9	9	9
1227	1.11E-05	9	9	9
1228	6.67E-05	9	9	9
1230	2.22E-05	9	9	9
1231	2.22E-05	9	9	9
1238	6.67E-05	9	9	9
1242	1.11E-04	9	9	9
1248	1.11E-05	9	9	9
1250	8.89E-05	9	9	9
1254	1.11E-05	9	9	9
1258	1.11E-05	9	9	9
1259	1.11E-05	9	9	9
1260	1.56E-04	9	9	9
1265	2.22E-05	9	9	9
1267	1.11E-05	9	9	9
1274	2.22E-05	9	9	9
1275	1.11E-05	9	9	9
1276	4.44E-05	9	9	9
1279	4.44E-05	9	9	9
1280	8.89E-05	9	9	9
1281	1.11E-04	9	9	9
1285	3.33E-05	9	9	9
1286	2.22E-05	9	9	9
1287	1.11E-05	9	9	9
1288	2.22E-05	9	9	9
1290	1.11E-05	9	9	9
1294	1.11E-05	9	9	9
1295	1.11E-05	9	9	9
1298	5.56E-05	9	9	9
1303	2.22E-05	9	9	9
1304	2.22E-05	9	9	9
1306	2.22E-05	9	9	9
1309	1.11E-05	9	9	9
1311	1.33E-04	9	9	9
1312	2.22E-05	9	9	9
1313	3.33E-05	9	9	9
1314	2.22E-05	9	9	9
1315	4.44E-05	9	9	9
1317	1.11E-05	9	9	9
1318	4.44E-05	9	9	9

1319	3.56E-04	9	9	9
1321	3.33E-05	9	9	9
1322	1.11E-05	9	9	9
1323	1.11E-05	9	9	9
1325	2.22E-05	9	9	9
1326	8.89E-05	9	9	9
1327	3.33E-05	9	9	9
1328	7.78E-05	9	9	9
1329	1.11E-05	9	9	9
1332	1.11E-05	9	9	9
1334	3.33E-05	9	9	9
1335	7.78E-05	9	9	9
1336	1.11E-05	9	9	9
1337	4.44E-05	9	9	9
1342	4.44E-05	9	9	9
1344	1.11E-05	9	9	9
1345	2.22E-05	9	9	9
1346	1.78E-04	9	9	9
1347	2.22E-05	9	9	9
1348	2.22E-05	9	9	9
1351	3.33E-05	9	9	9
1352	2.22E-05	9	9	9
1355	3.33E-05	9	9	9
1356	2.22E-05	9	9	9
1357	2.22E-05	9	9	9
1358	4.44E-05	9	9	9
1359	2.22E-05	9	9	9
1361	3.33E-05	9	9	9
1363	2.56E-04	9	9	9
1364	3.33E-05	9	9	9
1365	2.22E-05	9	9	9
1366	2.22E-05	9	9	9
1367	1.11E-05	9	9	9
1370	1.00E-04	9	9	9
1371	4.44E-05	9	9	9
1373	7.78E-05	9	9	9
1375	1.78E-04	9	9	9
1376	1.11E-05	9	9	9
1380	1.00E-04	9	9	9
1382	1.11E-05	9	9	9
1383	1.11E-05	9	9	9
1384	1.11E-05	9	9	9
1387	4.44E-05	9	9	9

1390	2.22E-05	9	9	9
1391	2.22E-05	9	9	9
1392	2.22E-05	9	9	9
1393	3.33E-05	9	9	9
1395	3.33E-05	9	9	9
1396	3.33E-05	9	9	9
1397	4.44E-05	9	9	9
1398	5.56E-05	9	9	9
1401	1.11E-05	9	9	9
1403	2.22E-05	9	9	9
1404	5.56E-05	9	9	9
1405	1.11E-05	9	9	9
1407	3.33E-05	9	9	9
1408	2.22E-05	9	9	9
1413	4.44E-05	9	9	9
1415	3.33E-05	9	9	9
1416	4.44E-05	9	9	9
1417	1.11E-05	9	9	9
1426	2.22E-05	9	9	9
1427	2.22E-05	9	9	9
1428	1.11E-05	9	9	9
1429	1.11E-05	9	9	9
1430	3.00E-04	9	9	9
1431	2.67E-04	9	9	9
1434	8.89E-05	9	9	9
1436	2.22E-05	9	9	9
1439	5.56E-05	9	9	9
1441	4.44E-05	9	9	9
1446	1.11E-05	9	9	9
1449	1.11E-05	9	9	9
1450	1.11E-05	9	9	9
1452	1.78E-04	9	9	9
1453	2.22E-05	9	9	9
1455	1.11E-05	9	9	9
1460	4.44E-05	9	9	9
1463	1.33E-04	9	9	9
1465	2.22E-05	9	9	9
1466	6.67E-05	9	9	9
1470	2.22E-05	9	9	9
1472	3.11E-04	9	9	9
1478	2.22E-05	9	9	9
1479	3.33E-05	9	9	9
1482	3.33E-05	9	9	9

1483	1.11E-05	9	9	9
1484	1.11E-05	9	9	9
1485	1.11E-05	9	9	9
1486	1.78E-04	9	9	9
1488	1.00E-04	9	9	9
1490	1.33E-04	9	9	9
1491	7.78E-05	9	9	9
1492	2.22E-05	9	9	9
1494	4.44E-05	9	9	9
1496	6.67E-05	9	9	9
1497	7.78E-05	9	9	9
1498	1.11E-05	9	9	9
1499	2.22E-05	9	9	9
1500	1.11E-05	9	9	9
1501	1.11E-05	9	9	9
1502	2.67E-04	9	9	9
1505	3.33E-04	9	9	9
1506	1.78E-04	9	9	9
1507	7.78E-05	9	9	9
1508	2.22E-05	9	9	9
1509	3.33E-05	9	9	9
1510	2.22E-05	9	9	9
1512	2.22E-05	9	9	9
1513	1.11E-05	9	9	9
1515	1.11E-05	9	9	9
1516	2.67E-04	9	9	9
1517	3.78E-04	9	9	9
1518	1.11E-05	9	9	9
1520	2.22E-05	9	9	9
1524	4.44E-05	9	9	9
1525	3.33E-05	9	9	9
1527	2.22E-05	9	9	9
1529	8.89E-05	9	9	9
1530	3.33E-05	9	9	9
1534	1.56E-04	9	9	9
1535	1.11E-05	9	9	9
1537	4.44E-05	9	9	9
1538	3.33E-05	9	9	9
1541	1.11E-05	9	9	9
1542	1.11E-04	9	9	9
1545	3.56E-04	9	9	9
1547	2.22E-05	9	9	9
1549	3.33E-05	9	9	9

1550	1.11E-05	9	9	9
1555	3.33E-05	9	9	9
1557	2.22E-05	9	9	9
1558	1.11E-04	9	9	9
1560	3.33E-04	9	9	9
1561	2.22E-05	9	9	9
1563	8.89E-05	9	9	9
1565	3.33E-05	9	9	9
1568	1.33E-04	9	9	9
1569	1.11E-05	9	9	9
1570	3.33E-05	9	9	9
1574	1.11E-05	9	9	9
1575	1.44E-04	9	9	9
1577	4.44E-05	9	9	9
1579	2.67E-04	9	9	9
1580	1.11E-05	9	9	9
1581	1.89E-04	9	9	9
1583	1.11E-05	9	9	9
1586	2.22E-05	9	9	9
1587	2.22E-05	9	9	9
1588	6.67E-05	9	9	9
1590	1.11E-05	9	9	9
1591	1.33E-04	9	9	9
1595	2.22E-05	9	9	9
1596	4.44E-05	9	9	9
1597	2.22E-05	9	9	9
1598	4.44E-05	9	9	9
1600	2.22E-05	9	9	9
1601	8.89E-05	9	9	9
1606	4.44E-05	9	9	9
1607	4.44E-05	9	9	9
1608	4.44E-05	9	9	9
1610	7.78E-05	9	9	9
1611	1.11E-05	9	9	9
1612	3.33E-05	9	9	9
1613	4.44E-05	9	9	9
1614	3.33E-05	9	9	9
1618	1.78E-04	9	9	9
1619	1.11E-05	9	9	9
1623	3.33E-05	9	9	9
1624	1.11E-05	9	9	9
1626	2.22E-05	9	9	9
1628	1.11E-05	9	9	9

1630	5.56E-05	9	9	9
1631	4.44E-05	9	9	9
1634	1.11E-05	9	9	9
1636	2.67E-04	9	9	9
1638	2.22E-05	9	9	9
1640	2.22E-05	9	9	9
1642	3.33E-05	9	9	9
1643	2.22E-05	9	9	9
1645	4.44E-05	9	9	9
1647	7.78E-05	9	9	9
1648	2.22E-05	9	9	9
1649	3.33E-05	9	9	9
1651	2.22E-05	9	9	9
1655	1.11E-05	9	9	9
1656	1.11E-05	9	9	9
1657	3.33E-05	9	9	9
1662	3.33E-05	9	9	9
1663	2.22E-04	9	9	9
1664	4.44E-05	9	9	9
1667	7.78E-05	9	9	9
1668	4.44E-05	9	9	9
1670	3.33E-05	9	9	9
1671	1.11E-05	9	9	9
1672	1.11E-05	9	9	9
1673	1.11E-05	9	9	9
1674	1.11E-05	9	9	9
1677	8.89E-05	9	9	9
1679	2.00E-04	9	9	9
1681	4.44E-05	9	9	9
1682	1.11E-05	9	9	9
1683	1.11E-05	9	9	9
1686	8.89E-05	9	9	9
1687	2.22E-05	9	9	9
1688	8.89E-05	9	9	9
1690	1.00E-04	9	9	9
1692	1.11E-05	9	9	9
1694	1.11E-05	9	9	9
1697	3.33E-05	9	9	9
1698	8.89E-05	9	9	9
1699	1.11E-05	9	9	9
1700	2.22E-05	9	9	9
1701	6.67E-05	9	9	9
1702	4.44E-05	9	9	9

1706	1.11E-05	9	9	9
1707	1.11E-05	9	9	9
1708	1.11E-05	9	9	9
1709	8.89E-05	9	9	9
1710	8.89E-05	9	9	9
1712	8.89E-05	9	9	9
1716	3.33E-05	9	9	9
1720	3.33E-05	9	9	9
1721	3.33E-05	9	9	9
1723	8.89E-05	9	9	9
1724	3.56E-04	9	9	9
1729	8.89E-05	9	9	9
1731	1.11E-05	9	9	9
1732	1.11E-05	9	9	9
1733	3.33E-05	9	9	9
1736	1.11E-05	9	9	9
1737	1.11E-05	9	9	9
1739	3.33E-05	9	9	9
1740	8.89E-05	9	9	9
1741	1.11E-05	9	9	9
1743	1.78E-04	9	9	9
1745	4.44E-05	9	9	9
1746	5.56E-05	9	9	9
1749	3.33E-05	9	9	9
1750	2.22E-05	9	9	9
1751	3.56E-04	9	9	9
1752	3.33E-05	9	9	9
1753	1.78E-04	9	9	9
1754	2.22E-05	9	9	9
1755	4.44E-05	9	9	9
1756	1.11E-05	9	9	9
1757	1.78E-04	9	9	9
1760	1.11E-05	9	9	9
1764	1.00E-04	9	9	9
1765	4.44E-05	9	9	9
1766	1.11E-05	9	9	9
1771	2.67E-04	9	9	9
1772	1.11E-05	9	9	9
1776	1.11E-05	9	9	9
1777	4.44E-05	9	9	9
1779	2.22E-05	9	9	9
1780	1.11E-04	9	9	9
1781	1.11E-05	9	9	9

1782	4.44E-05	9	9	9
1784	3.33E-05	9	9	9
1785	1.11E-05	9	9	9
1787	1.11E-04	9	9	9
1788	2.22E-05	9	9	9
1790	2.22E-05	9	9	9
1791	1.11E-05	9	9	9
1792	4.44E-05	9	9	9
1796	1.11E-05	9	9	9
1797	3.33E-05	9	9	9
1798	1.11E-05	9	9	9
1799	1.11E-05	9	9	9
1805	2.22E-05	9	9	9
1809	2.22E-05	9	9	9
1810	6.67E-05	9	9	9
1813	2.22E-05	9	9	9
1815	4.44E-05	9	9	9
1816	4.44E-05	9	9	9
1817	5.56E-05	9	9	9
1819	1.11E-05	9	9	9
1821	1.11E-05	9	9	9
1822	8.89E-05	9	9	9
1823	8.89E-05	9	9	9
1824	1.00E-04	9	9	9
1825	3.33E-05	9	9	9
1826	2.22E-05	9	9	9
1829	3.33E-05	9	9	9
1831	2.22E-04	9	9	9
1832	4.44E-05	9	9	9
1835	1.22E-04	9	9	9
1837	3.33E-05	9	9	9
1838	1.11E-05	9	9	9
1841	4.44E-05	9	9	9
1842	1.11E-05	9	9	9
1844	5.56E-05	9	9	9
1845	2.22E-05	9	9	9
1846	3.33E-05	9	9	9
1847	1.11E-04	9	9	9
1850	1.11E-05	9	9	9
1851	1.11E-05	9	9	9
1853	2.22E-05	9	9	9
1854	4.44E-05	9	9	9
1856	4.44E-05	9	9	9

1858	4.44E-05	9	9	9
1859	4.44E-05	9	9	9
1860	1.11E-05	9	9	9
1861	2.22E-05	9	9	9
1863	2.67E-04	9	9	9
1866	2.22E-05	9	9	9
1867	1.11E-05	9	9	9
1868	2.22E-05	9	9	9
1870	6.67E-04	9	9	9
1872	4.44E-05	9	9	9
1874	5.56E-05	9	9	9
1877	1.11E-05	9	9	9
1878	1.11E-05	9	9	9
1879	1.11E-05	9	9	9
1880	4.44E-05	9	9	9
1882	2.22E-05	9	9	9
1884	1.11E-05	9	9	9
1885	3.33E-05	9	9	9
1890	8.89E-05	9	9	9
1892	7.78E-05	9	9	9
1895	2.22E-05	9	9	9
1897	1.11E-05	9	9	9
1899	3.33E-05	9	9	9
1900	3.56E-04	9	9	9
1901	1.78E-04	9	9	9
1903	1.11E-05	9	9	9
1908	8.89E-05	9	9	9
1909	1.22E-04	9	9	9
1911	4.44E-05	9	9	9
1912	1.11E-05	9	9	9
1914	2.22E-05	9	9	9
1915	3.33E-05	9	9	9
1920	1.11E-05	9	9	9
1926	2.22E-05	9	9	9
1927	1.11E-04	9	9	9
1930	2.22E-05	9	9	9
1932	2.22E-05	9	9	9
1933	2.22E-05	9	9	9
1934	1.11E-05	9	9	9
1937	2.22E-05	9	9	9
1940	1.11E-05	9	9	9
1943	2.22E-05	9	9	9
1944	1.11E-05	9	9	9

1946	1.11E-05	9	9	9
1949	2.22E-05	9	9	9
1950	2.22E-05	9	9	9
1952	2.22E-05	9	9	9
1953	1.11E-05	9	9	9
1954	1.11E-05	9	9	9
1955	1.11E-05	9	9	9
1956	2.22E-05	9	9	9
1957	3.33E-05	9	9	9
1963	1.11E-05	9	9	9
1964	3.33E-05	9	9	9
1965	3.33E-05	9	9	9
1967	1.11E-05	9	9	9
1969	1.11E-05	9	9	9
1970	4.44E-05	9	9	9
1971	4.44E-05	9	9	9
1972	3.33E-05	9	9	9
1979	1.78E-04	9	9	9
1982	2.22E-05	9	9	9
1983	2.22E-05	9	9	9
1987	1.11E-05	9	9	9
1988	3.33E-05	9	9	9
1989	7.78E-05	9	9	9
1991	6.67E-05	9	9	9
1993	1.11E-05	9	9	9
1995	5.56E-05	9	9	9
1996	7.78E-05	9	9	9
1998	1.11E-05	9	9	9
1999	1.00E-04	9	9	9
2002	3.33E-05	9	9	9
2003	1.11E-05	9	9	9
2005	1.11E-05	9	9	9
2006	1.11E-05	9	9	9
2007	2.22E-05	9	9	9
2014	4.44E-05	9	9	9
2015	3.33E-05	9	9	9
2028	1.11E-05	9	9	9
2035	4.44E-05	9	9	9
2036	8.89E-05	9	9	9
2038	5.56E-05	9	9	9
2041	3.33E-05	9	9	9
2043	1.44E-04	9	9	9
2044	4.44E-05	9	9	9

2047	3.33E-05	9	9	9
2049	1.11E-05	9	9	9
2050	1.11E-05	9	9	9
2051	5.56E-05	9	9	9
2054	2.22E-05	9	9	9
2056	1.11E-05	9	9	9
2057	5.56E-05	9	9	9
2058	1.11E-05	9	9	9
2061	4.44E-05	9	9	9
2064	2.22E-05	9	9	9
2074	1.67E-04	9	9	9
2075	2.78E-04	9	9	9
2076	2.67E-04	9	9	9
2077	8.89E-05	9	9	9
2078	1.78E-04	9	9	9

Area	Mean	Min	Max
5.163	8.902	6	93
1.285	8.652	6	28
1.45	8.082	6	16
0.273	6.621	6	15
0.261	6.03	6	13
0.006	6.528	6	13
0.053	6.703	6	12
0.165	6.02	6	11
0.125	6.041	6	10
0.005	6.554	6	10
0.001	7.368	6	10
0.004	6.635	6	9
0.025	6.306	6	9
0.005	6.402	6	9
0.036	6.211	6	9
0.003	6.628	6	9
0.002	6.725	6	9
0.125	6.004	6	8
0.001	6.264	6	8
0.001	6.424	6	8
0.001	6.237	6	8
0.03	6.315	6	8
0.002	6.407	6	8
0.012	6.357	6	8
0.001	6.406	6	8
0.003	6.168	6	7
9.67E-04	6.172	6	7
0.002	6.267	6	7
0.001	6.109	6	7
0.002	6.132	6	7
0.002	6.023	6	7
0.001	6.039	6	7
6.22E-04	6.054	6	7
8.11E-04	6.027	6	7
9.78E-04	6.034	6	7
7.78E-04	6.043	6	7
9.44E-04	6.035	6	7
9.67E-04	6.034	6	7
9.56E-04	6.035	6	7
9.56E-04	6.023	6	7
0.001	6.122	6	7
7.33E-04	6.061	6	7

8.78E-04	6.051	6	7
0.019	6.025	6	7
7.00E-04	6.048	6	7
0.005	6.051	6	7
0.005	6.286	6	7
0.001	6.056	6	7
6.78E-04	6.049	6	7
5.56E-04	6.04	6	7
7.67E-04	6.217	6	7
5.67E-04	6.039	6	7
9.78E-04	6.182	6	7
7.89E-04	6.085	6	7
0.006	6.142	6	7
6.89E-04	6.048	6	7
0.001	6.186	6	7
0.002	6.248	6	7
0.002	6.145	6	7
0.022	6.003	6	7
0.001	6.084	6	7
0.001	6.154	6	7
6.22E-04	6.071	6	7
0.003	6.104	6	7
5.89E-04	6.057	6	7
0.001	6.189	6	7
8.67E-04	6.205	6	7
8.67E-04	6.205	6	7
0.001	6.208	6	7
0.009	6	6	6
1.00E-04	6	6	6
6.00E-04	6	6	6
6.78E-04	6	6	6
6.89E-04	6	6	6
7.78E-05	6	6	6
0.015	6	6	6
1.22E-04	6	6	6
6.67E-05	6	6	6
4.00E-04	6	6	6
3.33E-05	6	6	6
6.44E-04	6	6	6
9.33E-04	6	6	6
6.33E-04	6	6	6
3.11E-04	6	6	6
7.44E-04	6	6	6

6.67E-04	6	6	6
1.11E-04	6	6	6
2.22E-05	6	6	6
2.22E-04	6	6	6
0.052	6	6	6
2.22E-05	6	6	6
1.11E-05	6	6	6
7.78E-05	6	6	6
6.56E-04	6	6	6
0.004	6	6	6
3.00E-04	6	6	6
0.002	6	6	6
6.78E-04	6	6	6
6.89E-04	6	6	6
6.78E-04	6	6	6
6.56E-04	6	6	6
2.78E-04	6	6	6
4.56E-04	6	6	6
6.67E-05	6	6	6
5.22E-04	6	6	6
0.003	6	6	6
6.67E-04	6	6	6
6.89E-04	6	6	6
2.89E-04	6	6	6
6.11E-04	6	6	6
4.33E-04	6	6	6
3.22E-04	6	6	6
0.004	6	6	6
6.33E-04	6	6	6
8.89E-05	6	6	6
6.33E-04	6	6	6
1.11E-05	6	6	6
3.22E-04	6	6	6
6.44E-04	6	6	6
6.33E-04	6	6	6
6.89E-04	6	6	6
7.78E-05	6	6	6
7.00E-04	6	6	6
2.78E-04	6	6	6
7.67E-04	6	6	6
0.188	6	6	6
2.89E-04	6	6	6
4.33E-04	6	6	6

6.78E-04	6	6	6
8.89E-05	6	6	6
7.78E-05	6	6	6
3.22E-04	6	6	6
7.89E-04	6	6	6
7.11E-04	6	6	6
6.67E-04	6	6	6
6.78E-04	6	6	6
0.002	6	6	6
0.028	6	6	6
0.005	6	6	6
6.67E-04	6	6	6
6.67E-04	6	6	6
6.67E-05	6	6	6
2.22E-05	6	6	6
2.22E-05	6	6	6
5.56E-05	6	6	6
4.44E-05	6	6	6
5.56E-05	6	6	6
1.00E-04	6	6	6
1.11E-04	6	6	6
5.11E-04	6	6	6
4.56E-04	6	6	6
6.89E-04	6	6	6
5.00E-04	6	6	6
1.56E-04	6	6	6
1.11E-04	6	6	6
3.00E-04	6	6	6
1.11E-05	6	6	6
2.89E-04	6	6	6
3.33E-05	6	6	6
1.11E-04	6	6	6
8.89E-05	6	6	6
2.22E-05	6	6	6
3.33E-05	6	6	6
1.11E-05	6	6	6
1.11E-05	6	6	6
1.11E-05	6	6	6
1.11E-05	6	6	6
5.56E-05	6	6	6
3.44E-04	6	6	6
4.67E-04	6	6	6
0.001	6	6	6

1.11E-05	6	6	6
1.11E-05	6	6	6
3.33E-05	6	6	6
2.89E-04	6	6	6
2.78E-04	6	6	6
6.33E-04	6	6	6
6.67E-04	6	6	6
6.89E-04	6	6	6
3.11E-04	6	6	6
1.11E-05	6	6	6
9.89E-04	6	6	6
4.67E-04	6	6	6
1.44E-04	6	6	6
1.33E-04	6	6	6
4.89E-04	6	6	6
1.11E-05	6	6	6
0.004	6	6	6
6.78E-04	6	6	6
2.22E-05	6	6	6
6.89E-04	6	6	6
5.44E-04	6	6	6
0.007	6	6	6
6.89E-04	6	6	6
6.33E-04	6	6	6
2.78E-04	6	6	6
1.11E-05	6	6	6
6.89E-04	6	6	6
0.004	6	6	6
1.44E-04	6	6	6
7.11E-04	6	6	6
0.004	6	6	6
6.67E-05	6	6	6
6.56E-04	6	6	6
0.008	6	6	6
7.33E-04	6	6	6
0.006	6	6	6
6.22E-04	6	6	6
1.33E-04	6	6	6
3.11E-04	6	6	6
0.004	6	6	6
0.013	6	6	6
6.33E-04	6	6	6
0.001	6	6	6

7.44E-04	6	6	6
7.22E-04	6	6	6
4.67E-04	6	6	6
4.56E-04	6	6	6
0.013	6	6	6
3.33E-05	6	6	6
0.003	6	6	6
0.001	6	6	6
1.11E-05	6	6	6
2.22E-04	6	6	6
0.011	6	6	6
7.33E-04	6	6	6
2.78E-04	6	6	6
6.22E-04	6	6	6
0.003	6	6	6
6.89E-04	6	6	6
2.78E-04	6	6	6
2.33E-04	6	6	6
3.11E-04	6	6	6
