# Supplementary material for: Primary cilia control cell alignment and patterning in bone development via ceramide-PKCζ-β-catenin signaling
Source: Commun Biol. 2020 Jan 27;3:45. doi: 10.1038/s42003-020-0767-x (PMC6985158; doi:10.1038/s42003-020-0767-x)
Supplement: Supplementary file 11 — Reporting Summary [file 42003_2020_767_MOESM11_ESM.pdf]

nature research

Corresponding author(s): Shuying Yang

Last updated by author(s): Dec 6, 2019

Reporting Summary

Nature Research wishes to improve the reproducibility of the work that we publish. This form provides structure for consistency and transparency in reporting. For further information on Nature Research policies, see Authors & Referees and the Editorial Policy Checklist.

Statistics

For all statistical analyses, confirm that the following items are present in the figure legend, table legend, main text, or Methods section.

n/a	Confirmed	
☐	☒	The exact sample size (n) for each experimental group/condition, given as a discrete number and unit of measurement
☐	☒	A statement on whether measurements were taken from distinct samples or whether the same sample was measured repeatedly
☐	☒	The statistical test(s) used AND whether they are one- or two-sided<br>*Only common tests should be described solely by name; describe more complex techniques in the Methods section.*
☒	☐	A description of all covariates tested
☒	☐	A description of any assumptions or corrections, such as tests of normality and adjustment for multiple comparisons
☐	☒	A full description of the statistical parameters including central tendency (e.g. means) or other basic estimates (e.g. regression coefficient) AND variation (e.g. standard deviation) or associated estimates of uncertainty (e.g. confidence intervals)
☐	☒	For null hypothesis testing, the test statistic (e.g. F, t, r) with confidence intervals, effect sizes, degrees of freedom and P value noted<br>*Give P values as exact values whenever suitable.*
☒	☐	For Bayesian analysis, information on the choice of priors and Markov chain Monte Carlo settings
☒	☐	For hierarchical and complex designs, identification of the appropriate level for tests and full reporting of outcomes
☒	☐	Estimates of effect sizes (e.g. Cohen's d, Pearson's r), indicating how they were calculated

Our web collection on statistics for biologists contains articles on many of the points above.

Software and code

Policy information about availability of computer code

Data collection	Microscope software platform LAS X were used for immunofluorescent imaging.
Data analysis	Excel statistic analysis or online free student t-test:<br>https://www.socscistatistics.com/tests/studentttest/default2.aspx

For manuscripts utilizing custom algorithms or software that are central to the research but not yet described in published literature, software must be made available to editors/reviewers. We strongly encourage code deposition in a community repository (e.g. GitHub). See the Nature Research guidelines for submitting code & software for further information.

Data

Policy information about availability of data

All manuscripts must include a data availability statement. This statement should provide the following information, where applicable:

- Accession codes, unique identifiers, or web links for publicly available datasets
- A list of figures that have associated raw data
- A description of any restrictions on data availability

We provide a list of figures that are with associated raw data.

Field-specific reporting

Please select the one below that is the best fit for your research. If you are not sure, read the appropriate sections before making your selection.

☒ Life sciences ☐ Behavioural & social sciences ☐ Ecological, evolutionary & environmental sciences

Life sciences study design

All studies must disclose on these points even when the disclosure is negative.

Sample size	Sample size were determined to be either n=3 or n>3.
Data exclusions	Data were excluded when the internal controls were not showing correct outcome. For example, when actin was not showing even loading of the samples in Western analysis; or when the dapi staining were not showing good nuclei staining in immunofluorescence.
Replication	All the experimental results reported in this study was reproducible. The results that were not reproducible had NOT been written in the manuscript.
Randomization	The sequence of mice to be sacrificed or fixed, dissected or processed were randomized in our experimental procedures.
Blinding	Blinding was not possible because sample collecting and analysis have been performed by the same person.

Reporting for specific materials, systems and methods

We require information from authors about some types of materials, experimental systems and methods used in many studies. Here, indicate whether each material, system or method listed is relevant to your study. If you are not sure if a list item applies to your research, read the appropriate section before selecting a response.

Materials & experimental systems

n/a	Involved in the study	
☐	☒	Antibodies
☒	☐	Eukaryotic cell lines
☒	☐	Palaeontology
☐	☒	Animals and other organisms
☒	☐	Human research participants
☒	☐	Clinical data

Methods

n/a	Involved in the study	
☒	☐	ChIP-seq
☒	☐	Flow cytometry
☒	☐	MRI-based neuroimaging

Antibodies

Antibodies used

b-Actin Antibody (C4) HRP (1:2000)(Santa Cruz Biotechnology sc-47778 HRP),
Rabbit polyclonal anti-Arl13b(1:100 for immunofluorescence) (Proteintech 17711-1-AP),
Mouse monoclonal Anti-Ceramide (1:100 for immunofluorescence) (Sigma-Aldrich C8104-50TST),
pPKCz H-2 (Santa Cruz Biotechnology sc-271962),
PKCzH-1(Santa Cruz Biotechnology sc-17781),
CDC42B-8 (Santa Cruz Biotechnology sc-8401),
phospho-GSK3b F-2 (Santa Cruz Biotechnology sc-373800),
phospho-GSK3a/b 6D3 (Santa Cruz Biotechnology sc-81496),
GSK3b E-11 (Santa Cruz Biotechnology sc-377213),
b-catenin E-5 (Santa Cruz Biotechnology sc-7963),
Monoclonal Rat anti-PerlecanA7L6 (Santa Cruz Biotechnology sc-33707)

Validation

All the antibodies were either validated by Western analysis to show correct molecular weight OR validated by immunofluorescence method to show correct staining pattern such as correct sub-cellular localizations in our lab. In addition, these antibodies have also been used in other studies with the references provided by manufacturers.
e.g. (1) Ceramide: https://www.sigmaaldrich.com/catalog/product/sigma/c8104?lang=en&region=TW
(2) Arl13b: https://www.ptglab.com/products/ARL13B-Antibody-17711-1-AP.htm
(3) pPKCz: https://www.scbt.com/p/p-pkc-zeta-antibody-h-2
(4) PKCz: https://www.scbt.com/p/pkc-zeta-antibody-h-1
(5) CDC42: https://www.scbt.com/p/cdc42-antibody-b-8
(6) b-catenin: https://www.scbt.com/p/beta-catenin-antibody-e-5
(7) pGSK3-b: https://www.scbt.com/p/p-gsk-3beta-antibody-f-2
(8) pGSK3-a/b: https://www.scbt.com/p/p-gsk-3alpha-beta-antibody-6d3
(9) GSK3-b: https://www.scbt.com/p/gsk-3beta-antibody-e-11
(10) Perlecan: https://www.scbt.com/p/perlecan-antibody-a7l6

Animals and other organisms

Policy information about studies involving animals; ARRIVE guidelines recommended for reporting animal research

Laboratory animals

1. C57B6 neonatal mice were used for primary osteoblasts culture; 2. Mice with either OSX-cre transgenic or OSX-cre specific

Laboratory animals	IFT20 knockout of 3 months of age were sacrificed, fixed and analyzed for various different assays; 3. Mice with either IFT20 floxed or Col1-creERT IFT20 floxed allelles injected tamoxifen at postnatal day 4 and 6 were later on sacrificed at 1 month of age for further analysis. Some of the mice were sacrificed at postnatal day 14.
Wild animals	Did not involve wild animals.
Field-collected samples	Did not involve field-collected samples.
Ethics oversight	Did not involve field-collected samples.

Note that full information on the approval of the study protocol must also be provided in the manuscript.
